# Effectiveness of blended learning in pharmacy education: A systematic review and meta-analysis

**DOI:** 10.1371/journal.pone.0252461

**Published:** 2021-06-17

**Authors:** Athira Balakrishnan, Sandra Puthean, Gautam Satheesh, Unnikrishnan M. K., Muhammed Rashid, Sreedharan Nair, Girish Thunga

**Affiliations:** 1 Department of Pharmacy Practice, Manipal College of Pharmaceutical Sciences, Manipal Academy of Higher Education, Manipal, Udupi, Karnataka, India; 2 Department of Pharmacy Practice, National College of Pharmacy, Kozhikode, Kerala, India; 3 NGSM institute of Pharmaceutical Sciences, NITTE University, Manglore, Karnataka; National Taiwan University of Science and Technology, TAIWAN

## Abstract

**Background & objective:**

Though blended learning (BL), is widely adopted in higher education, evaluating effectiveness of BL is difficult because the components of BL can be extremely heterogeneous. Purpose of this study was to evaluate the effectiveness of BL in improving knowledge and skill in pharmacy education.

**Methods:**

PubMed/MEDLINE, Scopus and the Cochrane Library were searched to identify published literature. The retrieved studies from databases were screened for its title and abstracts followed by the full-text in accordance with the pre-defined inclusion and exclusion criteria. Methodological quality was appraised by modified Ottawa scale. Random effect model used for statistical modelling.

**Key findings:**

A total of 26 studies were included for systematic review. Out of which 20 studies with 4525 participants for meta-analysis which employed traditional teaching in control group. Results showed a statistically significant positive effect size on knowledge (standardized mean difference [SMD]: 1.35, 95% confidence interval [CI]: 0.91 to 1.78, p<0.00001) and skill (SMD: 0.68; 95% CI: 0.19 to 1.16; p = 0.006) using a random effect model. Subgroup analysis of cohort studies showed, studies from developed countries had a larger effect size (SMD: 1.54, 95% CI: 1.01 to 2.06), than studies from developing countries(SMD: 0.44, 95% CI: 0.23 to 0.65, studies with MCQ pattern as outcome assessment had larger effect size (SMD: 2.81, 95% CI: 1.76 to 3.85) than non-MCQs (SMD 0.53, 95% CI 0.33 to 0.74), and BL with case studies (SMD 2.72, 95% CI 1.86–3.59) showed better effect size than non-case-based studies (SMD: 0.22, CI: 0.02 to 0.41).

**Conclusion:**

BL is associated with better academic performance and achievement than didactic teaching in pharmacy education.

## Introduction

Evaluating the effectiveness of blended learning (BL), a thoughtful combination of both online and face-to-face instructions, is difficult because the components of BL can be extremely heterogeneous [1, 2]. For instance previous systematic reviews / meta-analyses on BL have included multiple techniques such as virtual face-to-face interaction, simulations, online instruction, e-mails, computer laboratories, mapping and scaffolding tools, computer clusters, interactive presentations, handwriting capture, class room web sites, and virtual apparatuses [3]. Also, there is no standardized proportion in which BL combines online with face-to-face instructions [4].

Flipped learning ‘and ‘hybrid learning’ are often used interchangeably with BL. In flipped learning, the learner is first exposed to online content, which will be reinforced during face-to-face sessions [5]. Hybrid learning, a combination of face-to-face instruction with computer mediated instruction, is most often used in United States [6]. In all forms of BL, the learner enjoys a certain degree of autonomy in deciding the pace of learning. However, previous reported systematic reviews on BL have not taken the keyword “flipped” in their search strategy [7, 8].

Increased research has been published on BL in medical education over last decades. For instance, Quian Liu et al’s systematic review and meta-analysis reported that BL has consistent positive effects in comparison with no intervention for knowledge acquisition in the health professions [7]. In another systematic review, McCutcheon et al reported a deficit of evidence on implementation of BL in undergraduate nursing education [9]. Most of the published systematic review and meta-analyses in medical education were focused on medical students or nursing students or other healthcare professionals [8–10]. There is only one meta-analysis that evaluated the effectiveness of flipped learning in pharmacy education, with a major limitation namely, lack of prospective randomized control trials (RCT) and restrictions to the domain of flipped contexts [11]. Accordingly, we designed our objective to assess the effectiveness of BL which employed a combination of online and face-to-face instruction in blended, hybrid and flipped contexts in pharmacy education. We have considered BL as a combination of online and face-to-face instruction, excluding other computer mediated forms like virtual labs, gamifications, simulations to limit heterogeneity and included all possible synonyms of blended, hybrid, flipped learning and pharmacy education.

## Materials and methods

This study followed Preferred Reporting Items for Systematic Reviews and Meta-Analyses (PRISMA) Guidelines (PRISMA Checklist attached in S1 Appendix).

### Eligibility criteria

We employed PICOS (population, intervention, comparison, outcome, and study design) framework for the inclusion of studies. Studies were considered eligible, if they: (1) were conducted among pharmacy students, (2) used a BL intervention in the experimental group, (3) used traditional lecture based learning as control for two arm studies and pre-test score for single arm studies (4) reported knowledge score/ objective structured clinical examination (OSCE) score as outcome (5) were two-group controlled studies (randomised/non-randomised)/ single group pre-test-post- test studies.

We excluded studies which did not explicitly state components of BL i.e. face-to-face learning and computer assisted learning. Computer assisted learning can be any form of technologies like online learning, e-learning, video podcasts, or the application of university learner management system for posting lectures. We excluded studies which employed “virtual face-to-face” interactions (as practiced by universities with satellite campuses). Studies which did not report a quantitative outcome of knowledge (comparison of students who completed and did not complete online module, number of correct answers between the groups, comparison of pass percentage), studies which evaluated only online component of BL, and surveys were also excluded. Multi-year studies without differentiating between study term years were excluded. Reviews, short communication, conference proceedings, editorials, meeting abstracts and non-English studies were also excluded.

### Data sources and literature search

A literature search employing PubMed, Scopus and Cochrane Library, was performed using a comprehensive search strategy since the inception of each database up to mid-December 2020. We employed all the MesH terms and key words for "BL" (Blended learning, blended course, blended program, hybrid learning, hybrid Course, Hybrid Program, Hybrid training, Flipped learning, Flipped Course, Flipped Program, Computer-aided learning, Computer-assisted learning, Integrated learning, Distributed learning, Distributed education Integrated instruction, Computer-aided instruction, Computer-assisted instruction) and “Pharmacy Student" which was obtained from the databases and previous studies. We employed the asterisk (*) as a wildcard character in keyword searches. We also searched for additional reference materials by consulting the cross references listed in the included publications, in addition to Google and Google Scholar (Details in S2 Appendix).

### Study selection and data extraction

The retrieved studies from databases were screened for its title and abstracts followed by the full-text in accordance with the pre-defined inclusion and exclusion criteria (List of excluded studies provided in S3 Appendix). We compiled and collated data in a comprehensive data extraction form containing characteristics such as, author and year of publication, population, duration and subject covered, nature of BL, sample size, and outcomes. The above data extraction form was perfected by trial and error, by piloting on 3 articles. Three independent reviewers were involved in study selection and data extraction to limit the bias and any disagreements were resolved through consensus or by discussion with another member of research team.

### Quality assessment

Modified Newcastle Ottawa scale (Newcastle Ottawa scale-education) was used to appraise methodological quality of included studies [12–14]. This tool assessed the following criteria: 1) representativeness of intervention group (1 point) 2) selection of comparison group (1point) 3) comparability of comparison group (2 point) 4) study retention (1 point) 5) blinding of assessment (1 point), totalling a maximum of 6 points. Two independent reviewers were involved to appraise the methodological quality to limit the bias and any disagreements were resolved through consensus or by discussion with another member of research team.

### Data synthesis

The evidence were synthesized narratively and presented in tabular form. We employed meta-analysis whenever possible. We omitted studies from data pooling whenever data did not meet the requirements of meta-analysis, such as, participant number, mean and standard deviation [SD]. All comparisons were based on scores of consecutive years. If more than one topic was delivered by BL in same study with separate scores for each, we considered them as separate studies. RevMan 5.3 was used to conduct the meta-analysis [15]. The data were used as mean with SD and outcomes were presented as standardised mean difference (SMD) along with 95% confidence interval (CI). Studies that did not report a SD, the corresponding SD from the p-values and standard errors were generated as per Cochrane guideline [16]. Heterogeneity was assessed by I^2^ statistics and random effect model used for statistical modelling. Subgroup analysis were performed to find out potential source of heterogeneity based on factors like studies with case studies and without case studies, studies which reported outcome as a measure of multiple choice questions(MCQs) or non MCQs, and studies from developed and developing countries. Sensitivity analysis were performed to ensure the robustness of findings.

### Publication bias

We employed a funnel plot for visual inspection of publication bias, which was assessed for statistical significance by Egger’s and Begg’s test [16].

## Results

**Fig 1:**
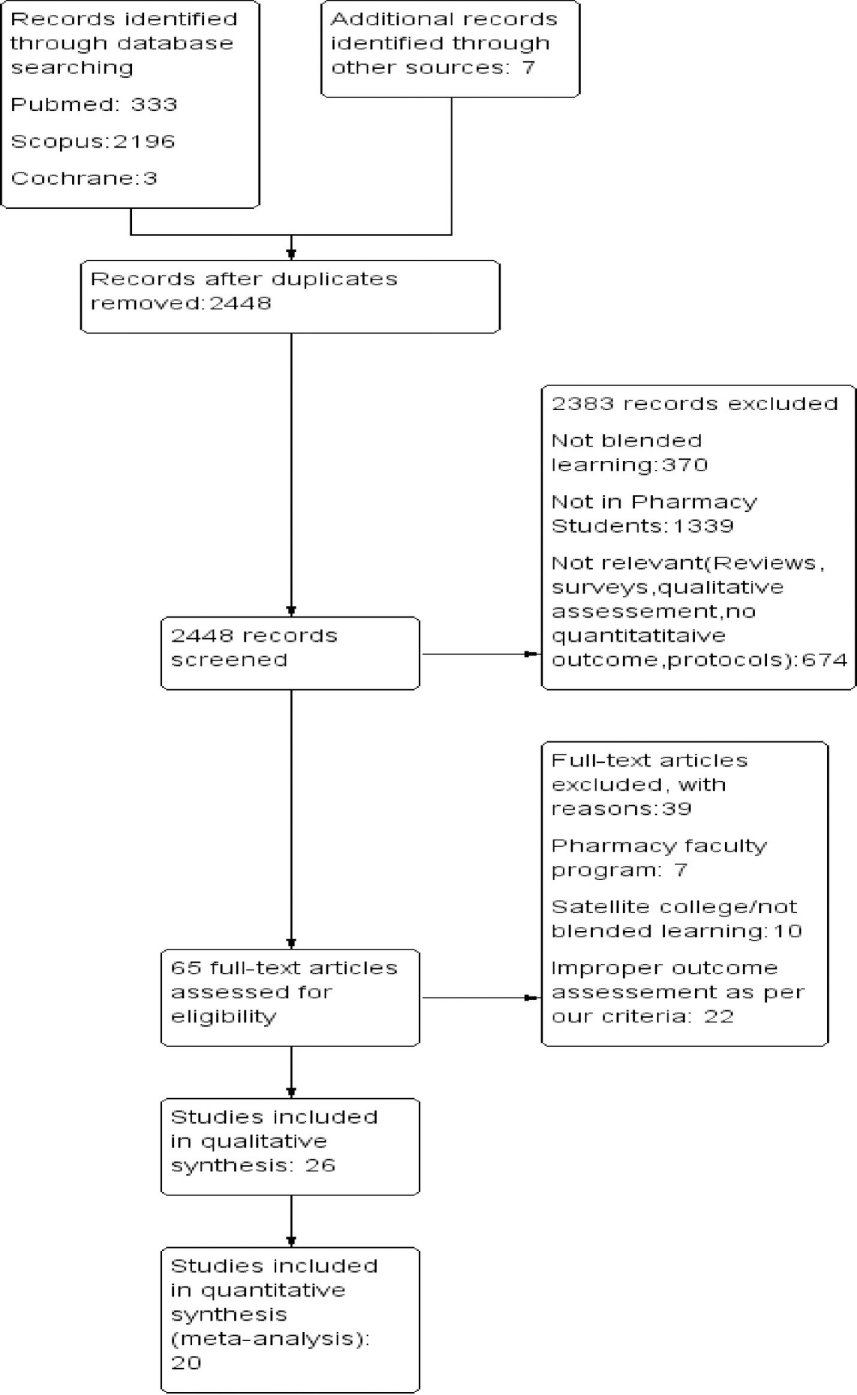
PRISMA flow diagram.

A total of 2539 records were retrieved first, of which 2448 underwent initial screening. Next, 2383 studies were omitted, yielding 65 full-text studies, of which 26 studies were included for systematic review, and 20 for meta-analysis (See Fig 1 for details of study selection).

### Characteristics of studies included for systematic review

Of the 26 studies included, only two employed single arm pre-test-post-test design [17, 18]. The remaining 24 studies were controlled studies [19–42] out of which 19 used examination scores of previous year [19–34, 36–41] and one used examination score of subsequent year as control [35]. There were 3 randomised trials [14, 19, 31] out of which one was cluster randomised [24]. Another study divided learning materials into didactic and BL in same population [28]. 18 studies originated from USA and 8 studies from other countries [17, 20, 21, 23–26, 28, 33] (See Table 1 for characteristics of included studies).

**Table 1:** Characteristics of included studies.

Author	Country	Population		Topic(duration)	Intrv. details	Study design	Sample size		Post-intrv. academic outcome	Other activities	Mean Result Score		Major outcome
							*Intrv*.	*Control*			*Intrv*.	*Control*	
Wilson et al [40] (2019)	USA	2^nd^ year pharmaco-therapy students		Selected self-care pharmaco-therapy(NC)	Online (Vimeo) + class activities	Cohort (compared with previous year students’ score)	N/A	N/A	Exam performance (65% course grade)-Assessment questions	TBL	83.5%	83.3%	No statistically significant differences in student outcomes
Newsom et al [34] (2019)	USA	1^st^ year students enrolled in spring 2015–17. (Control: spring ‘14)		Pharmacokinetics(NC)	Traditional class and video podcast	Cohort Intrv.: 2015–17 Didactic teaching: 2014	2015: 153	2014: 175	Final exam score: questions based on Bloom’s taxonomy.	Case based practice problems	2015: 85.8(7.7)	2014: 77.6 (13.3)	Final exam scores were significantly higher in spring ‘15 and ‘16 compared to ‘14 (p<0.001). 2017 scores were similar to that of 2014.
							2016: 152						
											2016: 85.1(9.2)		
							2017: 153						
											2017: 78(12)		
GoH et al [23] (2019)	Malaysia	2^nd^ year Dosage Form II course		3 credit course. Dosage form II(NC).	Pre-recorded video + F2F sessions	Cohort: Two group comparison (‘16 & ‘17 batch)	63	74	Final Exam score. Subjective (5 MCQ + 2 essay)	Online games	49.93	41.24	Final exam performance significantly higher in the flipped classroom group
He et al [24] (2019)	China	Junior year pharmacy under-graduates		Pharma-ceutical marketing (4 months)	Online + class	Cluster randomization	81	56	Final score (subjective–short answer, essays, MCQ)	Case discussion Group activity	88.21 ± 5.95	80.05 ± 5.59	Compared with LBL methods, implementing the FC model improved student performance.
Kouti et al [28] (2018)	Iran	Pharmacy Students (2015–16 batch)		Non-prescription drugs (1 semester)	Electronic based + lecture based	Propective comparative study– 3-group study (f2f, Electronic, BL)	57	-	Final exam score (not clear)	Case studies	E-learning group: 16.17 ± 0.33; Lecture group: 13.75 ± 0.16; BL: 16.39 ± 0.19	-	BL method and an e-learning approach can positively influence students’ knowledge towards non-prescription drugs
Kangwantas et al [26] (2017)	Thailand	2^nd^ year pharmacy students		Fundamental nutrition (1 year)	Videos (moodle platform)+class activities	Cohort compared with previous year score.	29	21	Pre and post-test within the group	Case discussion	Pre-test: (7.45±1.89 and Post-test: (8.17±1.44) not statistically different (p = 0.08).	Flipped class scored higher (7.24±1.24 vs. 6.19±1.76) (p = 0.028)	Student performance as measured by final scores of the module was better than those for the same module taught with a traditional lecture in previous year
									Post-test: main exam scores. (subjective-MCQ + short answer)				
Koo et al [27] (2016)	USA	2^nd^ year PharmD students		Pharmaco-therapy (1 year)	Online + F2F discussion	Cohort- comparison (2011 & 2012)	89	89	Exam score: Objective, MCQ	Case study discussion	88.2% (7.3%)	83.4% (7.9%)	The redesigned course improved student test performance and perceptions of learning experience
Giuliano et al [22] (2016)	USA	1^st^ year Pharmacy Students		Drug literature evaluation (1 year)	Youtube lecture +Class session.	Cohort: 2-group study (2013 & 2014)	94	99	Final exam score: subjective-application, analysis & Bloom’s taxonomy evaluation	Group activities	86.1%	75.6%	The flipped model is an excellent fit for drug literature content and courses that want to incorporate more active learning
Edginton et al [21] (2013)	Canada	2^nd^ year pharmacy students		Bio-chemistry (1 year)	online + classroom	Cohort: 2011 vs. 2010	116	109	Final grade: subjective-MCQ + calculation + long answer	Group discussion	78.8 + 11.7	61.8 + 17.8	The student driven BL model correlated positively with increased interest and perceived and actual learning gains. P<0.00001.
										Case studies			
Pierce [36] (2012)	USA	Pharmacy students		Renal pharmaco-therapy (8 weeks)	Video podcast + classroom	Pre-test and post-test-within the group (2012)	71	N/A	Objective-MCQ	Case discussion	Pre-test (33.5 ± 11.6 and post-test (79.2 ± 10.6): within the group	77.7 ± 4.7	Implementing a flipped classroom to teach renal pharmacotherapy resulted in improved student performance and favourable student perceptions
						Only post-test between groups (2012 & 2011)							
											Between groups: 81.6 ±4.4		
McLaughlin et al [31] (‎2015)	USA	PharmD students		Neuro-logic pharmaco-therapy(NC).	e-learning + class	Randomized (same class)	57	59	Final exam score: 9 final exam questions-not clear whether questions are MCQ/subjective.	Case studies	80.12 + 13.57	74.76 + 15.12	Interactive online preparatory tool improves student learning in neurologic pharmacotherapy.
Wong et al [41] (2014)	USA	1^st^ year pharmacy students		Cardiac arrhythmia (3 classes)	Pre-recorded video + class	Cohort: compared with previous year (2012 & 2011)	101	103	Final exam score: 5–6 MCQ on cardiac arrhythmias.	Case based exercises	Basic science: 88.3±1.9	84.1 ± 1.9; 56.8 ± 2.2; 73.7 ± 2.1	Use of the flipped teaching in a 3-class pilot on cardiac arrhythmias improved exam scores for pharmacology and therapeutics classes.
											Pharmacology: 89.6±2		
											Therapeutics: 89.2±1.4		
Anderson et al [19] (2017)	USA	1^st^ year pharmacy students		Pharma-ceutical calculations (6 weeks)	recorded lecture + video	Randomized	38	32	Final exam- Skill: OSCE Score at 6 weeks	Case studies	71.3 (14.7)%	61.8 (17.7)%	Average OSCE performance was be higher in flipped model than lecture model
Cotta et al [20] (2016)	Georgia	1^st^ year pharmacy students		Pharma-ceutical calculation (10 weeks)	Pre-recorded video + class	Cohort: 2012 vs. 2011	151	165	Final exam part 2 score-objective graded quizzes	-	88.3 (9.5)	84.1 (11.3)	Flipped classroom can improve student performance and satisfaction in pharmaceutical calculations (P<0.001)
Lancaster [29] (2011)	USA	2^nd^ PharmD		OTC, medicines (15 weeks)	Pre-recorded video + class	Cohort: 2008 vs. 2009	97	97	Final Exam scores-objective-9 quizzes	Clinical based cases with Group discussion	84.09	65.15	Students performed significantly higher on quizzes and examinations when using this hybrid teaching model.
										Puzzles,			
										Think pair share activities.			
Stewart [38] (2013)	USA	3^rd^ year pharmaco-therapy		Pharmaco-therapy(NC)	Podcast + active learning	Cohort: 2009 vs. 2010	71	65	Final exam score: 20 MCQ	Group discussion	72.9+1.5 (12.63)	77.15+1.2 (9.6)	The class averages on the final exams were significantly higher for 2009 compared to 2010 batch (P: 0.019).
Lockman et al [30] (2017)	USA		1^st^ year pharmaco-logy & therapeutics course	Pain manag-ement module(NC)	E learning + in class lecture	Cohort: 2015 vs. 2016	162	156	OSCE: skill	Cases & mini cases, quiz games,	MCQ: 82.30% (SD 10.25)	77.23% (SD 12.43	Student performance improved significantly after flipping the content of pain management module.
									Knowledge-MCQ				
											OSCE: 79.34(9)	OSCE: 67.01(9.6)	
										Mind-mapping debates			
Nazar et al [33] (2018)	Qatar		Stage 2 Pharmacy under-graduate students	Pharmacy law(NC)	Online class + In-class activities.	Cohort study: compared with previous year	69 (2016–17)	63 (2015–16)	Final summative examination score	Group discussion	82.2 (6.3)%	84.2 (6.8)%	Examination performance appeared to be unaffected by the change in teaching style
								37 (2014–15)				83.0 (7.6)%	
Hughes et al [25] (2016)	USA		P1 pharmacy students	Drug information (5 weeks)	Narrated video + F2F lab session	Cohort study: compared with previous years (2012 Vs. 2013)	127	121	Final exam score: Objective-40 MCQ	-	88.99%	84.87%	Mean final exam scores significantly increased (p < 0.05)
Gloudeman et al [42] (2017)	USA		1^st^ year pharmacy students	Pharmaceutical calculation (6 week)	Online video + classroom session	Cohort study: compared with previous years (2015 Vs. 2014)	102	104	Final exam score: 13 pharma-ceutical calculation questions	-	80.5 ± 15.8%	77.8 ± 16.8%	The mean exam scores of the intrv. were not significantly different than the control (p = 0.253)
Czepula et al [17] (2017)	Brazil		Under-graduate bachelor’s degree in pharmacy	Pharma-ceutical care(NC)	F2F + distance learning	Quasi-experimental, prospective.	Pharma-ceutical care 1: 82		Pre- and post-test: 30 MCQ	-	Module 1: Mean scores increased from 4.8 to 6.3 (p<0.05)		Positive results were observed regarding the students’ performance in the two disciplines
						Two groups. Both groups received BL of pharma-ceutical care 1 & 2.	Pharmaceutical care 2: 51						
											Module 2: Mean scores increased from 4.1 to 5.5 (p<0.05)		
Prescott et al [37] (2016)	USA		1^st^ and 2^nd^ year pharmacy students	Two course: PA1 and PA2(NC)	Online videos + class	Cohort study: Comparison of traditional and BL of PA1 & PA2	PA1: 130	PA1: 126	Final examination score: 20 short answer questions	Group discussion(TBL)	Knowledge: PA1: 80.5 (9.6)	Know-ledge: PA1: 73(12);	BL was associated with improved academic performance and was received by students.
							PA2: 131	PA2: 122					
											PA2: 80.6 (14.3)		
										Case based learning		PA2: 74.5(12.1)	
											P<0.001		
											Skill: PA1: 93.1 (7.6)		
						(2014–15 vs, 2013–14)							
											PA2: 83.5 (12.5)	P<0.001	
												Skill: PA1: 89.1 (13.8)	
												PA2: 81.5 (12.6)	
Wanat et al [39] (2016)	USA		3^rd^ year pharmacy students	Critical care 2hr credit course(4 weeks)	Video recorded lecture + in-class activities	Cohort study: Compared with previous year score. (2013, 2014 Vs. 2012, 2011)	51	54	Overall exam performance: subjective: online quiz + skills: examining patients	Group learning	87.7% (3.7)	82.6% (6.3)	Exam scores of students in BL group is significantly higher than control
Phillips et al [35] (2016)	USA		1^st^ and 2^nd^ year PharmD students	EBM & Therapeutics(6 month)	Online video prior to class room	Cohort study: Knowledge compared with previous year scores -two group comparison.	EBM: 201	N/A	Final Exam score/-not clear whether questions are MCQ/subjective.		EBM:83%	EBM:85%	Use of the BLE did not seem to have an impact on long-term knowledge in this study
							Therapeutics: 199				Therapeutics: 97		
												Therapeutics: 98%	
Hess et al [18] (2016)	USA		2^nd^ year pharmacy students	Patient centred communication skills (One semester,)	Online modules + small group discussion	Single group study	57	-	Pre-test and post-test	Group discussion	Significant Increase in scores from pre-test to post-test.7 domains of pre and posttest scores provided.		Patient-centred interprofessional communication skills improved significantly with BL
									OSCE				
McLaughlin et al [32] (2014)	USA		1^st^ year pharmaceutics students	Pharmaceutics course(NC)	Flipped classroom (iLAMs + F2F)	Cohort study: Traditional vs. Flipped	162	153	Final exam grade. Subjective-quizes+examination scores	-	Final score; 165.48 ± 13.34	160.06 ± 14.65	Higher final exam grades in flipped classroom
						2012 vs. 2011							

Intrv.: Intervention; BL: Blended learning, EBM: Evidence-based medicine; TBL: Team Based Learning; F2F: Face-to-Face, N/A-not Available, NC-not clear MCQ: Multiple choice questions, OSCE: Objective structured clinical examination, PA1: Patient assessment 1 course, PA2: Patient assessment 2 course, iLAMS.: integrated learning accelerator module.

### Outcome measured

Only 3 studies [18, 19, 39] reported outcome as skills(patient centred interpersonal communication skills, students’ performance on pharmaceutical calculation, and critical care therapeutics) while 21 studies reported only knowledge score [17, 20–29, 31–36, 38, 40–42]. Two reported both knowledge and skills as outcomes [30, 37]. Outcomes were measured variably as mean examination percentage (n = 16) or mean examination score (n = 6) or objective structured clinical examination (OSCE) (n = 2). Two studies reported both examination percentage and OSCE score.

### BL approaches

Two studies employed face-to-face session followed by online activities [17, 34] while all other studies employed face-to-face session after watching online content. Only one study reported time spent and workload associated with BL [37].

### Quality assessment of included studies

As per modified Ottawa scale requirements, we ascertained that intervention groups in all the included studies were representatives of target population. Out of 26 studies, 19 used previous year students’ score as control, one used subsequent year score as control and 3 studies were randomized. Two studies used analysis of covariance(ANCOVA) for controlling covariates in final analysis [23, 38] and one used linear regression [22]. In five studies there were no statistically significant differences in students demographics / pre-test (Grade Point Average) between groups by t-test [27, 30, 32, 34, 41]. However, modified Ottawa scale requires controlling for subject characteristics by statistical covariate analysis. Outcome assessment was blinded for 11 studies, as assessor cannot be influenced by group assessment (third party statistician) or assessments did not require human judgments (MCQs/ graded performance) [17, 19–20, 25, 27, 29–30, 36, 38, 40–41]. As all studies were part of curriculum in educational institution, there is no mention about drop outs. All studies obtained a score below 4 except one [19] (See S4 Appendix).

### Quantitative analysis

We included 20 studies with 4525 participants for meta-analysis that employed traditional teaching in the control group and had no missing data.

### Efficacy of BL versus. Traditional teaching in improving knowledge

**Fig 2:**
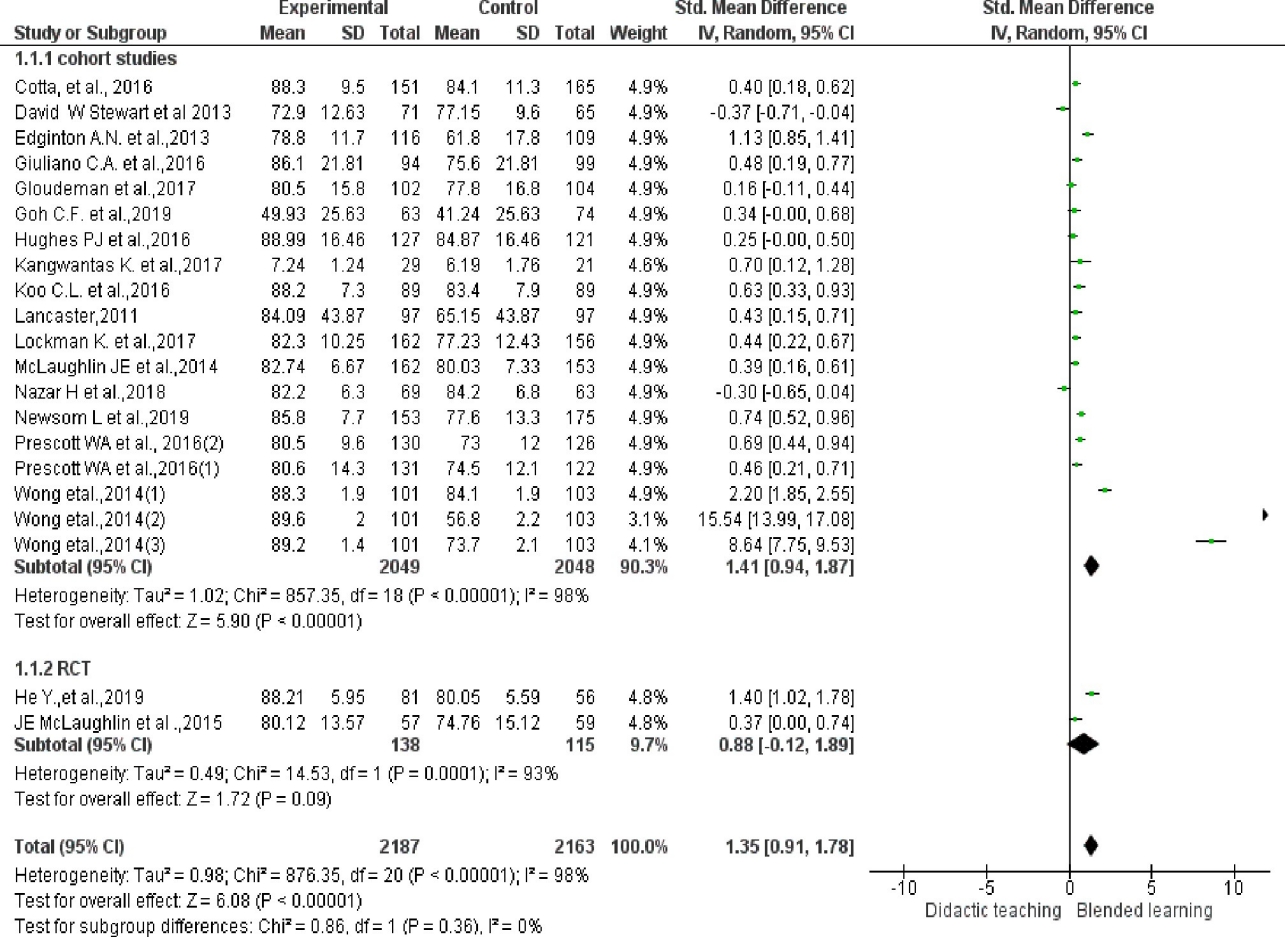
Efficacy of BL vs. traditional teaching in improving knowledge. If more than one topic was delivered by BL in same study (Prescott, Wong) with separate scores for each, we considered them as separate studies (Prescott 1&2, Wong 1, 2&3).

Pooled effect of 18 studies showed that knowledge improved significantly in BL, with large effect compared to didactic teaching ((SMD 1.35, 95% CI-0.91 to 1.78, p<0.00001). In the knowledge domain, randomised controlled studies had a lower pooled effect (SMD 0.88) than cohort studies (SMD 1.41). There was significant statistical heterogeneity among studies (I^2^ = 98%, p<0.00001) with individual effect sizes ranging from −0.37 to 15.54 (See Fig 2).

### Efficacy of BL versus traditional teaching in improving skill

**Fig 3:**
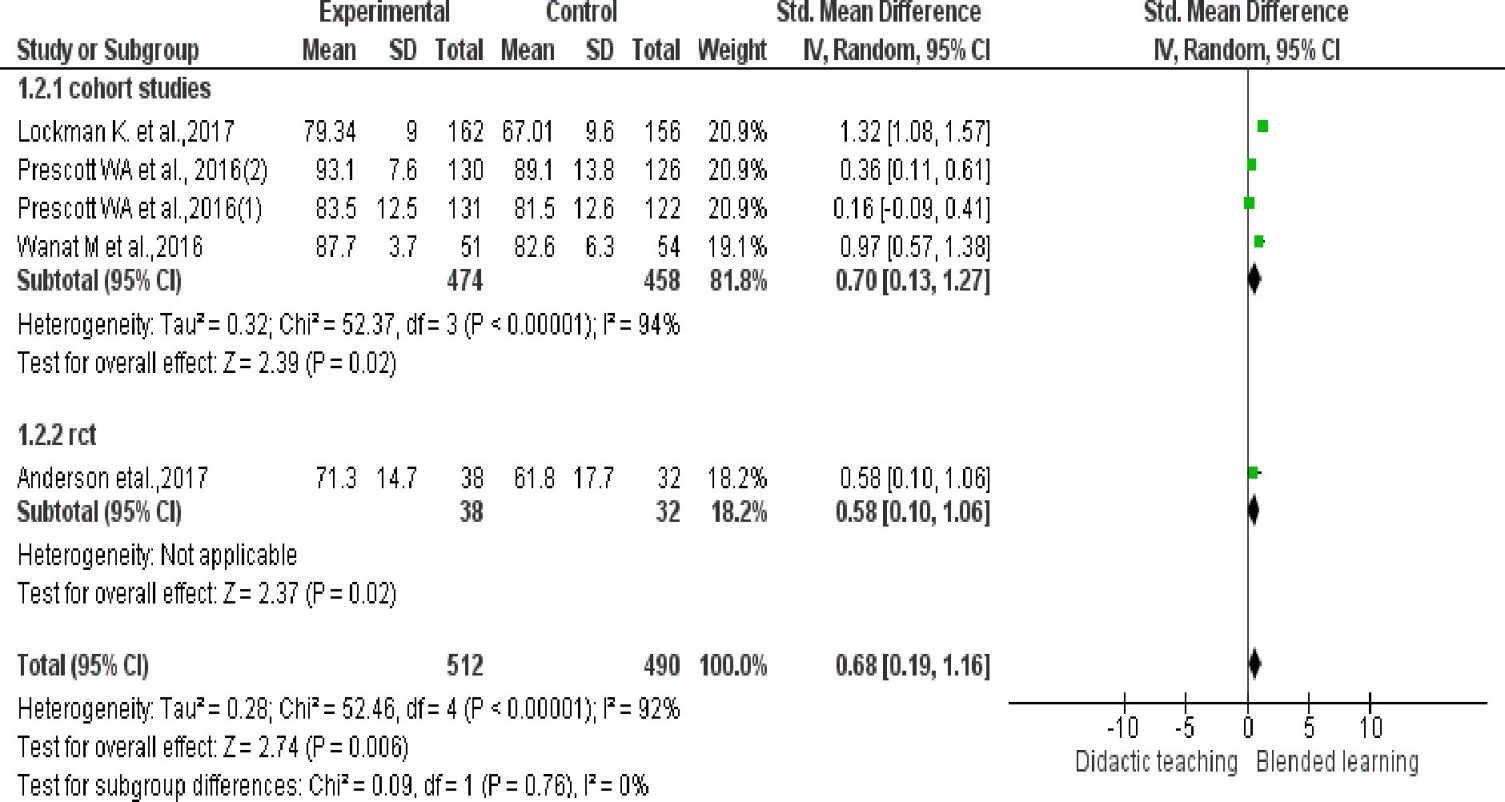
Efficacy of BL vs. traditional teaching in improving skill. If more than one topic was delivered by BL in same study (Prescott) with separate scores for each, we considered them as separate studies (Prescott 1&2).

Pooled effect size (SMD 0.68, 95% CI: 0.19 to 1.16,Z = 2.74,p = 0.006) of 4 studies in improving skills, showed statistically significant moderate to large effect, compared with didactic teaching. Significant statistical heterogeneity was observed among studies (I^2^ = 92%, p<0.00001) (See Fig 3).

### Subgroup analysis

Subgroup analysis of cohort studies, in the knowledge domain, demonstrated advantage for BL over traditional teaching, in developed countries (SMD 1.54, 95% CI 1.01–2.06) than developing countries (SMD 0.44, 95% CI 0.23–0.65). Studies which employed MCQ scores as outcome showed larger effect size (SMD 2.81, 95% CI 1.76–3.85) than non MCQs (SMD 0.53, 95% CI 0.33–0.74). Also, studies which employed case studies/case discussion favoured BL (SMD 2.72, 95% CI 1.86–3.59) than non-case based studies (SMD: 0.22, CI: 0.02 to 0.41). Subgroup analyses of studies improving skill were not performed, as all studies originated from United States of America and all employed case studies/case discussion. (See Table 2)

**Table 2:** Subgroup analysis of cohort studies.

Study Characteristics:	Sample size	Test for heterogeneity			Test for effect	
		I^2^(%)	Q statistics	P value	Pooled effect size(SMD(C1))	P value
1. Country						
Developed	3731	98	854.67	P<0.00001	1.54(1.01,2.06)	P<0.00001
Developing	366	0	0.89	P = 0.35	0.44(0.23,0.65)	P<0.0001
Total	4097	98	857.3	P<0.00001	1.41(0.94,1.87)	P<0.00001
2 Outcome assessment						
MCQ	2002	99	796.46	P<0.00001	2.81(1.76,3.85)	P<0.0001
Non MCQ	1635	76	29.47	P<0.0001	0.53(0.33,0.74	P<0.0001
Not clear	460	96	24.89	P<0.00001	0.23(-0.80,1.25)	0.66
Total	4097	98	857.35	P<0.00001	1.41(0.94,1.87)	P<0.00001
3. Case studies						
Present	2364	99	736.66	P<0.00001	2.72(1.86,3.59)	P<0.00001
Absent	1733	75	31.53	P<0.0001	0.22(0.02,0.41)	0.03
Total	4097	98	857.36	P<0.00001	1.41(0.94,1.87)	P<0.00001

MCQ: Multiple choice questions; SMD: Standardised mean difference; CI: confidence interval.

### Sensitivity analysis

**Fig 4:**
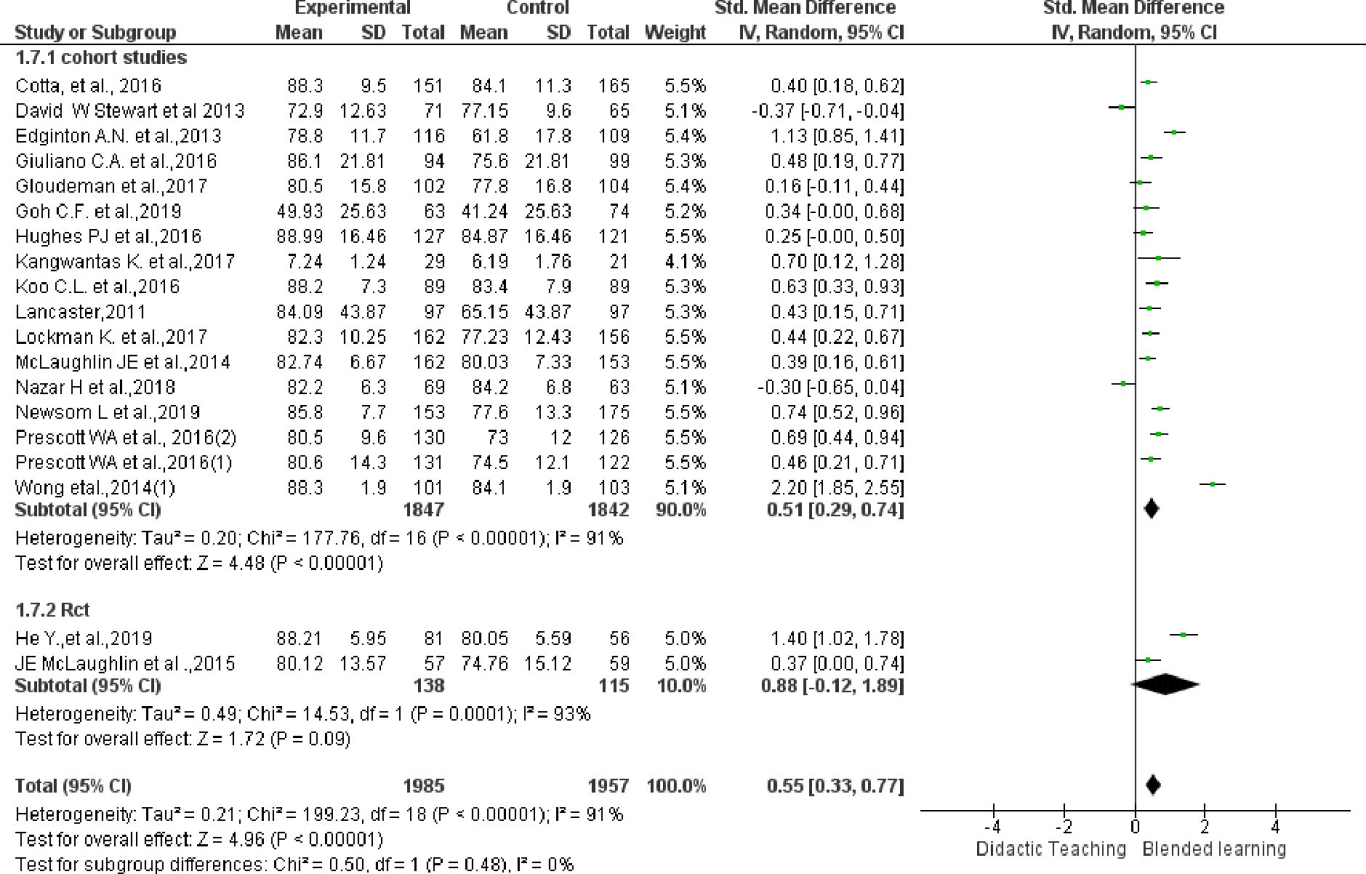
Sensitivity analysis: If more than one topic was delivered by BL in same study (Prescott, Wong) with separate scores for each, we considered them as separate studies (Prescott 1&2).

A sensitivity analysis was performed in studies improving knowledge by removing two studies (Wong et al., [2, 3]) which are having lesser weight (3.1% and 4.1%, respectively), and higher outlier (MD: 15.54 and 8.64, respectively) which supported the main results (SMD: 0.55; 95%CI: 0.33 to 0.77). The result of sensitivity analysis is depicted in Fig 4.

### Publication bias

**Fig 5:**
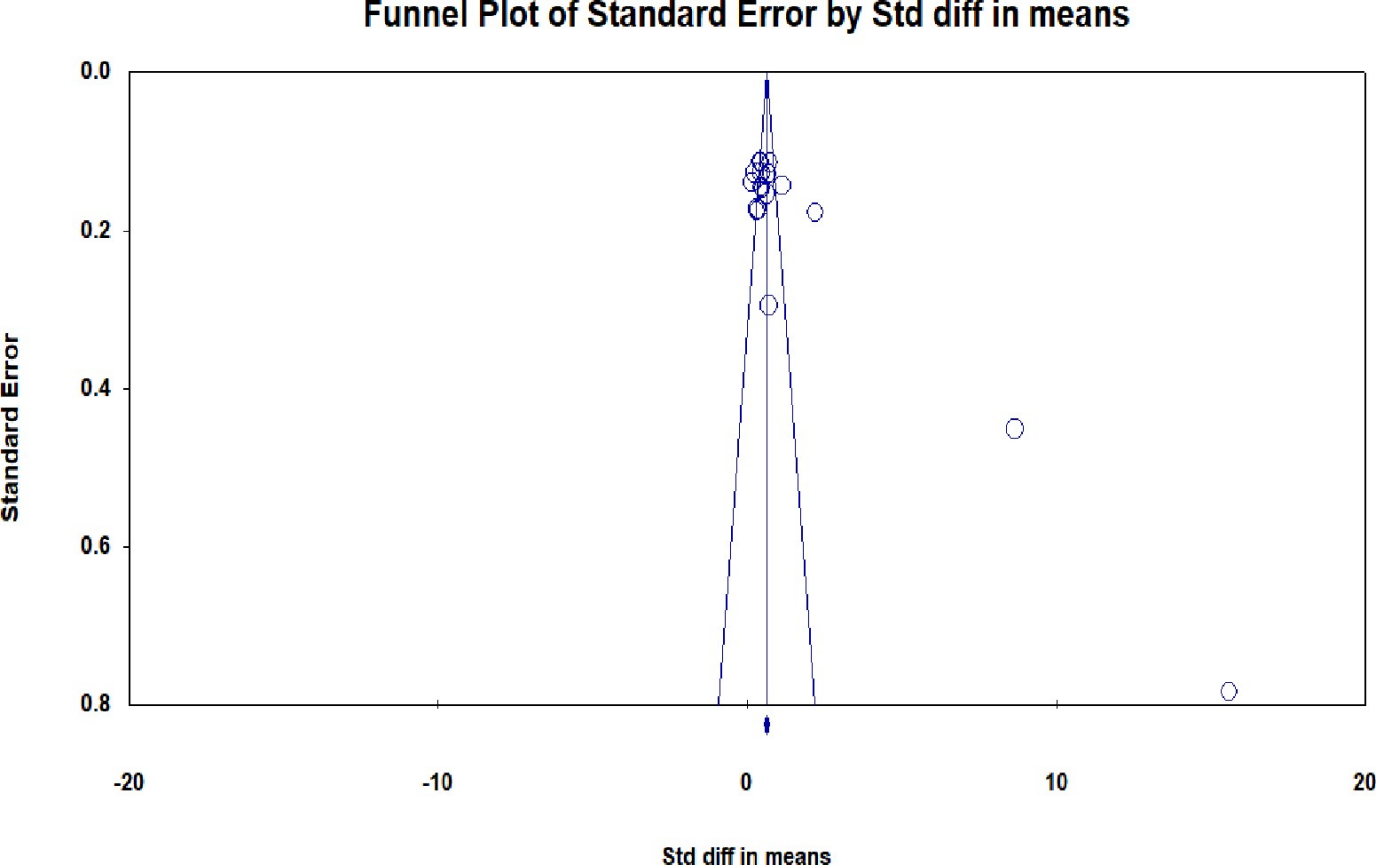
Funnel plot of BL versus traditional teaching in improving knowledge.

Visual inspection of funnel plot revealed an obvious asymmetry, demonstrating possible publication bias. This was confirmed by Egger’s (P = 0.00006) and Begg’s (P = 0.04) test (See Fig 5).

## Discussion

This systematic review and meta-analysis primarily attempted to evaluate the impact of BL approach on various outcomes in pharmacy education. We identified 26 studies relevant for systematic review, in which 18 demonstrated significant improvement in learning outcome, against controls. Two of them were single arm studies which also showed improved performance after intervention. 24 of the 26 studies included in this systematic review were controlled, among which majority (n = 19) employed examination scores of previous year(s) as the control. All studies employed first online review of contents followed by face-to-face discussion except two. Studies which employed face-to-face discussion followed by online activities also favoured BL [17, 34]. The face- to- face discussion part of BL in all included studies involved either reinforcing the concepts by tutor or using learning strategies such as case studies, case discussion or group activities.

In addition to the general scarcity of literature comparing BL and traditional methods, a major limitation of the previous meta-analysis by Gillette et al., was the lack of prospective RCTs [11]. Our meta-analysis included 20 of the studies included in the systematic review. Our review included 3 RCTs, all of which showed major improvements in either knowledge score or skill. We report a large pooled effect size for knowledge and a medium to large for skills. These findings were statistically significant with high heterogeneity in all analyses and are consistent with those reported by previous meta-analyses in medical education.

The majority of the studies reported knowledge score in terms of either mean examination percentage/score or OSCE, whereas 5 studies reported outcomes based on skill. Many of the studies included in this review also reports that BL has a major effect on improving teaching as well as positive student perceptions about learning. As mentioned earlier, the rich variety of components can attribute to an enhanced learning experience as well as increased engagement and learning activities such as group assessment, assessment quizzes and peer discussions. Even the studies that did not report a significant difference in acquisition of knowledge–such as those by Phillips et al., and Gloudeman et al. showed that the perceptions of both students and faculty favoured BL [35, 42].

Another important finding is that BL modules which employed case studies/discussions or case-based scenarios reported better outcomes. A few studies also concluded that positive results obtained may not be attributed entirely to the suggest on that case studies need to be included in learning strategies [24, 37]. There is evidence to show that case studies simulate real world situations and enhance interactive student-centred learning, particularly in the health professions. Incorporating case studies in a real-world context is extensively useful in pharmacy education, as it enhances students’ complex decision-making abilities.

Out of 26 studies, only 4 originated from developing countries, possibly because of poor online connectivity, lack of resources, fear of adopting unfamiliar technology, lack of skill development program to instructors, interruption in power supply and internet connections, affordability, low bandwidth and trust deficit [17, 20, 26, 28]. A single study that compares time budgets reported that BL techniques were completed ahead of allotted time [35]. BL approach appears to significantly improve the learning outcomes in pharmacy students and reason could be following,

- **Relaxed/flexible scheduling**: BL allows students to view electronic materials at their own pace and time
- **Improved interaction**: BL makes classroom discussion more meaningful because of content familiarity.
- **Variety of components**: BL incorporates a rich variety of face-to-face and online components.

This study has a few limitations. First, the search was restricted to the publications in English language, which might have contributed to missing out eligible studies in non-English speaking countries. However, a comprehensive search in various databases would have covered the maximum quality publications. Second, our review also excludes conference proceeding and unpublished or grey literature. However, this may increase the credibility of our findings obtained from full length papers by avoiding the irrelevant or incomplete acquisition of the data. Third, there was high heterogeneity among the outcomes or measures of outcome, thereby restricting our choice exclusively to studies reporting quantitative outcomes. Fourth, the heterogeneous administration pattern of BL was an another challenge in this review, so we included those studies which used online teaching along with face-to-face approach, this made our result more robust and conclusive. Statistical heterogeneity was high in all analysis. However, this is in accordance with other meta-analysis in medical education [7, 8, 43]. Subgroup analyses did not find any source of heterogeneity. Despite the effective search strategy, one major limitation is the majority i.e. 18 of the 26 studies, were from the US, which could impact the global representativeness of the findings. Therefore, future research should address the impact of BL in diverse populations from other countries.

Publication bias was addressed by including the three major scientific databases (Pubmed, SCOPUS and Cochrane) during the literature search. This resulted in an increased number of papers which may have further increased the likelihood of selecting papers with negative results. In our review, 5 of the 26 studies reported that BL yields either equal or poorer outcomes than didactic teaching [33, 35, 38, 40, 42].

## Conclusion

BL is associated with better academic performance and achievement than didactic teaching in pharmacy education. The COVID-19 pandemic is radically reshaping the education sector to transform from conventional teaching to more online learning. In this scenario, it is critical to conduct more controlled empirical studies to evaluate the effectiveness of BL. Such research can inform education policies and guidelines to standardise blended learning.

## Supporting information

S1 Appendix PRISMA checklist. (DOCX)

S2 Appendix Search strategy in database. (DOCX)

S3 Appendix List of excluded studies. (DOCX)

S4 Appendix Quality assessment. (DOCX)

## References

[1] Graham CR. Emerging practice and research in blended learning. In Moore M. G.(Ed.), Handbook of distance education. New York: Routledge.2013:333–350

[2] Dziuban C et al. Blended learning: the new normal and emerging technologies. ETHE. 2018; 15(1): 3.

[3] Means B et al. The effectiveness of online and blended learning: A meta-analysis of the empirical literature. *Teach*. *Coll*. *Rec*. 2013;115(3): 1–47.

[4] Garnham C. Introduction to hybrid courses. Teaching Technology Today. 2002;8.

[5] DeLozier SJ et al. Flipped classrooms: a review of key ideas and recommendations for practice. *Educ*. *Psychol*. *Rev*. 2017; 29(1):141–151.

[6] O’Byrne WI et al. Hybrid and blended learning: Modifying pedagogy across path, pace, time, and place. *J Adolesc Adult Lit*. 2015;59(2):137–140.

[7] Liu Q et al. The effectiveness of blended learning in health professions: systematic review and meta-analysis. *J Med Internet Res*. 2016;18(1): e2. doi: 10.2196/jmir.4807 26729058 PMC4717286

[8] Vallée A, Blacher J, Cariou A, Sorbets E. Blended Learning Compared to Traditional Learning in Medical Education: Systematic Review and Meta-Analysis. *J Med Internet Res*. 2020;22(8):e16504 doi: 10.2196/16504 32773378 PMC7445617

[9] McCutcheon K et al. A systematic review evaluating the impact of online or blended learning vs. face-to-face learning of clinical skills in undergraduate nurse education. *J Adv Nurs*. 2015;71(2):255–270. doi: 10.1111/jan.12509 25134985

[10] Rowe M, Frantz J, Bozalek V. The role of blended learning in the clinical education of healthcare students: a systematic review. *Medical teacher*. 2012;34(4):e216–21 doi: 10.3109/0142159X.2012.642831 22455712

[11] Gillette C et al. A Meta-Analysis of Outcomes Comparing Flipped Classroom and Lecture. *Am J Pharm Educ*.2018; 82(5):6898. doi: 10.5688/ajpe6898 30013248 PMC6041496

[12] Cook DA et al. Method and reporting quality in health professions education research: a systematic review. *Med Educ*. 2011;45(3):227–238. doi: 10.1111/j.1365-2923.2010.03890.x 21299598

[13] Cook DA et al. Internet-based learning in the health professions: a meta-analysis. *JAMA*. 2008; 300(10):1181–1196. doi: 10.1001/jama.300.10.1181 18780847

[14] Cook DA et al. Appraising the quality of medical education research methods: The Medical Education Research Study Quality Instrument and the Newcastle-Ottawa Scale-Education. *Acad Med*. 2015;90(8):1067–1076. doi: 10.1097/ACM.0000000000000786 26107881

[15] Collaboration, T. C. (2014). Review Manager. Copenhagen.

[16] Higgins JP et al. Cochrane handbook for systematic reviews of interventions: John Wiley & Sons.2019.

[17] dos Santos Czepula AI et al. Active methodology and blended learning: An experience in pharmaceutical care. *Curr Pharm Teach Learn*. 2018;10(1):106–111. doi: 10.1016/j.cptl.2017.09.013 29248067

[18] Hess R et al. Teaching communication skills to medical and pharmacy students through a blended learning course. *Am J Pharm Educ*. 2016;80(4). doi: 10.5688/ajpe80464 27293231 PMC4891862

[19] Anderson HG et al. Comparison of pharmaceutical calculations learning outcomes achieved within a traditional lecture or flipped classroom andragogy. *Am J Pharm Educ*. 2017;81(4). doi: 10.5688/ajpe81470 28630511 PMC5468708

[20] Cotta KI et al. Effectiveness of flipped classroom instructional model in teaching pharmaceutical calculations. *Curr Pharm Teach Learn*. 2016;8(5):646–653.

[21] Edginton AN et al. Using student feedback to design a more effective clinical biochemistry course component. *Curr Pharm Teach Learn*. 2013;5(1):23–32.

[22] Giuliano CA et al. Evaluation of a flipped drug literature evaluation course. *Am J Pharm Educ*. 2016;80(4). doi: 10.5688/ajpe80466 27293233 PMC4891864

[23] Goh CF et al. Flipped classroom as an effective approach in enhancing student learning of a pharmacy course with a historically low student pass rate. *Curr Pharm Teach Learn*. 2019; 11(6):621–629. doi: 10.1016/j.cptl.2019.02.025 31213319

[24] He Y et al. The effects of flipped classrooms on undergraduate pharmaceutical marketing learning: A clustered randomized controlled study. *PLOS One*. 2019;14(4), e0214624. doi: 10.1371/journal.pone.0214624 30969976 PMC6457546

[25] Hughes P. J., Waldrop B., Chang J. J. C. i. P. T., & Learning. (2016). Student perceptions of and performance in a blended foundational drug information course. *Curr Pharm Teach Learn*. 2016; 8(3):359–363. doi: 10.1016/j.cptl.2016.02.013 30070246

[26] Kangwantas K et al. Implementing a flipped classroom approach to a course module in fundamental nutrition for pharmacy students. *Pharm. Educ*. 2017; 17.

[27] Koo CL et al. Impact of flipped classroom design on student performance and perceptions in a pharmacotherapy course. *Am J Pharm Educ*. 2016;80(2). 10.5688/ajpe80233 PMC4827584 27073286

[28] Kouti L et al. Comparison of the effectiveness of three educational methods (e-learning, lectures and blended) on pharmacy students’ knowledge of non-prescription drugs. *Pharm. Educ*. 2018; 18:197–201.

[29] Lancaster JW et al. Online lecture delivery paired with in class problem-based learning… Does it enhance student learning? *Curr Pharm Teach Learn*.2011;3(1):23–29.

[30] Lockman K et al. Improved learning outcomes after flipping a therapeutics module: results of a controlled trial. *Acad Med*. 2017;92(12):1786–1793. doi: 10.1097/ACM.0000000000001742 28562458

[31] McLaughlin JE et al. Comparison of an interactive e-learning preparatory tool and a conventional downloadable handout used within a flipped neurologic pharmacotherapy lecture. *Curr Pharm Teach Learn*. 2015;7(1):12–19.

[32] McLaughlin JE et al. The flipped classroom: a course redesign to foster learning and engagement in a health professions school. *Acad Med*. 2014;89(2):236–243. doi: 10.1097/ACM.0000000000000086 24270916

[33] Nazar H et al. A study to investigate the impact of a blended learning teaching approach to teach pharmacy law. *Int J Pharm Pract*. 2019;27(3):303–310. doi: 10.1111/ijpp.12503 30548898

[34] Newsom L et al. Implementation and evaluation of problem-based video podcasts in an introductory pharmacokinetics course. *Curr Pharm Teach Learn*. 2019; 11(12):1213–1220. doi: 10.1016/j.cptl.2019.09.003 31836145

[35] Phillips JA et al. Time spent, workload, and student and faculty perceptions in a blended learning environment. *Am J Pharm Educ*. 2016;80(6). 10.5688/ajpe806102 PMC5023973 27667839

[36] Pierce R et al. Vodcasts and active-learning exercises in a “flipped classroom” model of a renal pharmacotherapy module. *Am J Pharm Educ*. 2012;76(10). 10.5688/ajpe7610196 PMC3530058 23275661

[37] Prescott WA et al. Introduction and assessment of a blended-learning model to teach patient assessment in a doctor of pharmacy program. *Am J Pharm Educ*. 2016;80(10). 10.5688/ajpe8010176 PMC5289732 28179725

[38] Stewart DW et al. An analysis of student performance with podcasting and active learning in a pharmacotherapy module. *Curr Pharm Teach Learn*. 2013;5(6):574–579.

[39] Wanat MA et al. A critical care hybrid online elective course for third-year pharmacy students. *Am J Pharm Educ*.2016;80(9). doi: 10.5688/ajpe809154 28090103 PMC5221836

[40] Wilson JA et al. Flipped classroom versus a didactic method with active learning in a modified team-based learning self-care pharmacotherapy course. *Curr Pharm Teach Learn*. 2019;11(12):1287–1295. doi: 10.1016/j.cptl.2019.09.017 31836155

[41] Wong TH et al. Pharmacy students’ performance and perceptions in a flipped teaching pilot on cardiac arrhythmias. *Am J Pharm Educ*. 2014;78(10). 10.5688/ajpe7810185 PMC4315207 25657372

[42] Gloudeman MW et al. Use of condensed videos in a flipped classroom for pharmaceutical calculations: Student perceptions and academic performance. *Curr Pharm Teach Learn*. 2018;10(2):206–210. doi: 10.1016/j.cptl.2017.10.001 29706277

[43] Fontaine G, Cossette S, Maheu-Cadotte MA, Mailhot T, Deschênes MF, Mathieu-Dupuis G, et al. Efficacy of adaptive e-learning for health professionals and students: a systematic review and meta-analysis. *BMJ open*. 2019;9(8): e025252. doi: 10.1136/bmjopen-2018-025252 31467045 PMC6719835

