# Supplementary material for: Effectiveness of blended learning in pharmacy education: A systematic review and meta-analysis
Source: PLoS One. 2021 Jun 17;16(6):e0252461. doi: 10.1371/journal.pone.0252461 (PMC8211173; doi:10.1371/journal.pone.0252461)
Supplement: S2 Appendix — (DOCX) [file pone.0252461.s002.docx]

**S2 Appendix. Search strategy in database.**

1. Search strategy in PubMed

| **Search** | **Query** | **Items found** | **FACETS** |
| --- | --- | --- | --- |
| #1 | Search ("Blended learning" OR hybrid learning OR integrated learning OR computer-aided learning OR computer-assisted learning OR distributed learning OR hybrid training OR integrated training OR computer-aided training OR integrated education OR computer-aided education OR "computer-assisted education" OR "distributed education" OR integrated instruction OR computer-aided instruction OR "computer-assisted instruction" OR blended teaching OR integrated teaching OR computer-aided teaching OR computer-assisted teaching OR blended course OR hybrid course OR integrated course OR computer-assisted course) | 13665 | Intervention |
| #2 | Search (pharmac*[Title/Abstract]) OR Pharmacy students | 234442 | Population |
| #3 | Search (#1) AND (#2) | 345 | I+P |
| #4 | Search (compar* OR trial* OR evaluat* OR assess* OR effect* OR pretest* OR pre-test OR posttest* OR post-test OR preinterven* OR pre-intervention OR postinterven* OR post-intervention) | 15241901 | Study design |
| #5 | Search (#3) AND (#4) | 333 | I+P+S |

1. Search strategy in Cochrane

| **Search** | **Query** | **Items found** | **FACETS** |
| --- | --- | --- | --- |
| #1 | "Blended learning" OR "Hybrid learning" OR "Flipped learning" | 109 | Intervention |
| #2 | "Pharmac*" OR "Pharmacy students" OR "Student Pharmacy" | 228 | Population |
| #3 | Search (#1) AND (#2) | 3 | I+P |

1. Search strategy in Scopus

| **Search** | **Query** | **Items found** | **FACETS** |
| --- | --- | --- | --- |
| #1 | "Blended learning" OR "blended course" OR "Blended program" OR "hybrid learning" OR "hybrid Course" OR "Hybrid Program" OR "Flipped learning" OR "Flipped Course" OR "Flipped Program" | 31,115 | Intervention |
| #2 | "Pharmac*" OR "Pharmacy Student" OR "Student Pharmacy" OR "Pharma students" | 7,665,900 | Population |
| #3 | Search (#1) AND (#2) | 2196 | I+P |
