# Supplementary material for: Effectiveness of blended learning in pharmacy education: A systematic review and meta-analysis
Source: PLoS One. 2021 Jun 17;16(6):e0252461. doi: 10.1371/journal.pone.0252461 (PMC8211173; doi:10.1371/journal.pone.0252461)
Supplement: S3 Appendix — (DOCX) [file pone.0252461.s003.docx]

**S3 Appendix. List of excluded studies.**

1. An advanced cardiovascular pharmacotherapy course blending online and face-to-face instruction single group.
2. Combined Use of Online Tutorials and Hands-On Group Exercises in Bibliographic Instruction for Pharmacy Students-Assignements
3. The Impact of Blended Learning on Student Performance in a Cardiovascular Pharmacotherapy Course
4. The trilayer approach of teaching physiology, pathophysiology, and pharmacology concepts in a first-year pharmacy course: the TLAT model
5. Advanced Screencasting With Embedded Assessments in Pathophysiology and Therapeutics Course Modules
6. The Use of Virtual Laboratories and Other Web-based Tools in a Drug Assay Course
7. Learning outcomes and student preferences with flipped vs lecture/case teaching model in a block curriculum
8. Contributions of a blended learning based on peer evaluation for teaching drug-drug interactions to undergraduate pharmacy students
9. Development and evaluation of a hybrid course in clinical virology at a faculty of Pharma in France
10. A Blended Learning Experience for Teaching Microbiology
11. The use of social media as a tool to educate United Kingdom undergraduate pharmacy students about public health
12. Student perceptions of a flipped pharmacotherapy course
13. Use of wikis in pharmacy hybrid elective courses
14. A Blended learning approach to teaching basic pharmacokinetics and the significance of face-to-face interaction
15. How to provide feedback to students' learning - Assignment and feedback concept in the blended learning environment pharma2
16. Design and evaluation of a new national pharmacy internship program in Ireland
17. A novel online platform promotes asynchronous class preparation and thought transparency
18. Student perceptions of a modified flipped classroom model for accreditation in a pharmacotherapeutics course
19. Blended versus face-to-face: Comparing student performance in a therapeutics class
20. A longitudinal online interprofessional education experience involving family nurse practitioner students and pharmacy students
21. Use of virtual patients in an advanced therapeutics pharmacy course to promote active, patient-centered learning
22. Blended-learning for courses in pharmaceutical analysis
23. Impact of novel active-learning approaches through ibooks and gamification in a reformatted pharmacy course
24. Integration of a Community Pharmacy Simulation Program into a Therapeutics Course
25. Preparing pharmacy students to communicate effectively with adolescents
26. Pharmacy student engagement, performance, and perception in a flipped satellite classroom
27. Student perception and academic performance after implementation of a blended learning approach to a drug information and literature evaluation course
28. The use of social media as a tool to educate United Kingdom undergraduate pharmacy students about public health
29. Comparison of Two Lecture Delivery Platforms in a Hybrid Distance Education Program
30. Impact of Hybrid Delivery of Education on Student Academic Performance and the Student Experience
31. Medication Therapy Management Training Using Case Studies and the MirixaPro Platform
32. Integration of a Community Pharmacy Simulation Program into a Therapeutics Course
33. Flip My Class! A faculty development demonstration of a flipped-classroom
34. Does a blended learning environment suit advanced practice training for pharmacists in a Middle East setting?
35. Training of the pharmaceutical team to vitamin K antagonists (VKA) consultations: A blended-learning experience in hospital
36. Effectiveness of flipped classroom with Poll Everywhere as a teaching-learning method for pharmacy students
37. Flipping content to improve student examination performance in a pharmacogenomics course
38. Implementation of a flipped classroom model to teach psychopharmacotherapy to third-year Doctor of Pharmacy (PharmD) students
39. Addition of Care for Transgender-Related Patient Care into Doctorate of Pharmacy Curriculum: Implementation and Preliminary Evaluation
