# Supplementary material for: Effectiveness of blended learning in pharmacy education: A systematic review and meta-analysis
Source: PLoS One. 2021 Jun 17;16(6):e0252461. doi: 10.1371/journal.pone.0252461 (PMC8211173; doi:10.1371/journal.pone.0252461)
Supplement: S4 Appendix — (DOCX) [file pone.0252461.s004.docx]

**S4 Appendix. Quality assessment.**

| **Author** | **Represent-ativeness** | **Selection** | **Comparability** | | | | **Blinding** | **Follow up** | **Total score** |
| --- | --- | --- | --- | --- | --- | --- | --- | --- | --- |
|  |  |  | **Non-Randomized 2-groups** | | **RCT** | | *Was Outcome assessment blinded?* | *Low follow-up loss  (< 25%)* |  |
|  |  |  | *Controlled pre-test scores* | *Other baseline characteristics* | *Randomized* | *Allocation concealment* |  |  |  |
| Wilson et al (2019) | 1 | 0* | 0 | 0 |  |  | 1 (third party statistician) | 0 | **2** |
| Newsom et al (2019) | 1 | 0* | 0 (statistical difference not mentioned) | 0 |  |  | 0 | 0 | **1** |
| GoH et al (2019) | 1 | 0* | 1 (Pretest (ANCOVA)) |  |  |  | 0 | 0 | **2** |
| He et al (2019) | 1 | 1 |  |  | 1 (Pre-test: GPA) | 0 | 0 | 0 | **3** |
| Kouti et al (2018) | 1 | 1 (Traditional & BL topics were randomized in same population) | 0 | 0 |  |  | 0 | 0 | **2** |
| Kangwantas et al (2017) | 1 | 0* | 0 | 0 |  |  | 0 | 0 | **1** |
| Koo et al (2016) | 1 | 0* | 0 (GPA scores, age & gender details) | 0 |  |  | 1 (MCQ) | 0 | **2** |
| Giuliano et al (2016) | 1 | 0* | 1 (GPA,PCAT (linear regression)) | 0 |  |  | 0 | 0 | **2** |
| Edginton et al (2013) | 1 | 0* | 0 | 0 |  |  | 0 | 0 | **1** |
| Pierce (2012) | 1 | 0* | 0 (Pre-test within the group) |  |  |  | 1 (MCQ) | 0 | **2** |
| McLaughlin et al (‎2015) | 1 | 1 (same batch students) |  |  | 1 | 0 | 0 | 0 | **3** |
| Wong et al (2014) | 1 | 0* | 0 (GPA: t-test) | 0 (Age (t-test); gender (chi-square) |  |  | 1 (MCQ) | 0 | **2** |
| Anderson et al (2017) | 1 | 1 |  |  | 1 | 0 | 1 (assessed with Blackboard Learn course) | 0 | **4** |
| Cotta et al (2016) | 1 | 0* | 0 | 0 |  |  | 1 (MCQ) | 0 | **2** |
| Lancaster (2011) | 1 | 0* | 0 | 0 |  |  | 1 (MCQ, post-session quiz) | 0 | **2** |
| Stewart (2013) | 1 | 0* | 1 (GPA,PCAT (ANCOVA)) | 0 |  |  | 1 (MCQ) | 0 | **3** |
| Lockman et al (2017) | 1 | 0* | 0 (Adjusted PCAT, grade point(t-test)) | 0 (age-t test) |  |  | 1 (SOAP notes selection for blinded rescoring) | 0 | **2** |
| Nazar et al (2018) | 1 | 0* | 0 | 0 |  |  | 0 |  | **1** |
| Hughes et al (2016) | 1 | 0* | 0 | 0 |  |  | 1 (MCQ) | 0 | **2** |
| Gloudeman et al (2017) | 1 | 0* | 0 | 0 |  |  | 0 | 0 | **1** |
| Czepula et al (2017) | 1 | 0 (no control group) |  |  |  |  | 1 (MCQ) | 0 | **2** |
| Prescott et al (2016) | 1 | 0* | 0 | 0 |  |  | 0 | 0 | **1** |
| Wanat et al (2016) | 1 | 0* | 0 | 0 |  |  | 0 | 0 | **1** |
| Phillips et al (2016) | 1 | 0 (Compared with next year’s scores) | 0 | 0 |  |  | 0 | 0 | **1** |
| Hess et al (2016) | 1 | 0 (no control group) |  |  |  |  | 0 | 0 | **1** |
| McLaughlin et al (2014) | 1 | 0* | 0 (GPA &PCAT scores(t-test)) | 0 |  |  | 0 | 0 | **1** |

*Historical Group i.e. scores of previous year students, as control.
