# Supplementary material for: Effectiveness of blended learning in pharmacy education: A systematic review and meta-analysis
Source: PLoS One. 2021 Jun 17;16(6):e0252461. doi: 10.1371/journal.pone.0252461 (PMC8211173; doi:10.1371/journal.pone.0252461)
Supplement: Attachment — Submitted filename: PONE-D-20-37558_R1_reviewer_resubmission.pdf [file pone.0252461.s007.pdf]

PLOS ONE

Effectiveness of blended learning in pharmacy education: A systematic review and meta-analysis

--Manuscript Draft--

Manuscript Number:	PONE-D-20-37558R1
Article Type:	Research Article
Full Title:	Effectiveness of blended learning in pharmacy education: A systematic review and meta-analysis
Short Title:	Blended learning in Pharmacy education
Corresponding Author:	Girish Thunga<br>Manipal Academy of Higher Education<br>udupi, INDIA
Keywords:	blended learning; flipped learning; education; knowledge; Pharmacy; Systematic review; Teaching
Abstract:	Background & Objective: Though blended learning (BL), is widely adopted in higher education, evaluating effectiveness of BL is difficult because the components of BL can be extremely heterogeneous. Purpose of this study was to evaluate the effectiveness of BL in improving knowledge and skill in pharmacy education.<br>Methods: . PubMed/MEDLINE, Scopus and the Cochrane Library were searched to identify published literature. The retrieved studies from databases were screened for its title and abstracts followed by the full-text in accordance with the pre-defined inclusion and exclusion criteria. Methodological quality was appraised by modified Ottawa scale. Random effect model used for statistical modelling.<br>Key findings: A total of 26 studies were included for systematic review. Out of which 20 studies with 4525 participants for meta-analysis which employed traditional teaching in control group. Results showed a statistically significant positive effect size on knowledge (standardized mean difference [SMD]: 1.35, 95% confidence interval [CI]: 0.91 to 1.78, p<0.00001) and skill (SMD: 0.68; 95% CI: 0.19 to 1.16; p=0.006) using a random effect model. Subgroup analysis of cohort studies showed, studies from developed countries had a larger effect size (SMD: 1.54, 95% CI: 1.01 to 2.06), than studies from developing countries(SMD: 0.44, 95% CI: 0.23 to 0.65, studies with MCQ pattern as outcome assessment had larger effect size (SMD: 2.81, 95% CI: 1.76 to 3.85) than non-MCQs (SMD 0.53, 95% CI 0.33 to 0.74), and BL with case studies (SMD 2.72, 95% CI 1.86-3.59) showed better effect size than non-case-based studies (SMD: 0.22, CI: 0.02 to 0.41).<br>Conclusion: BL is associated with better academic performance and achievement than didactic teaching in pharmacy education.
Order of Authors:	Athira Balakrishnan
	Sandra Puthean
	Gautam Satheesh
	Unnikrishnan M K
	Muhammed Rashid
	Sreedharan Nair
	Girish Thunga
Response to Reviewers:	Reviewers comments:<br>Reviewer 1:<br>Abstract - You can remove "This review follows the PRISMA guideline." - PRISMA is a reporting standard, not a methodology. In the full text, you can certainly say your review adheres to the reporting standards recommended by the PRISMA statement-<br>Answer:<br>Corrected as per reviewer<br><br>2. Eligibility (Page 3) - Please clarify. You state in (3) "pre-test score for single arm

Powered by Editorial Manager® and ProduXion Manager® from Aries Systems Corporation

	studies" and (5) "were two groups" - so did you included single group papers or not? Based on results, you included 2 single arm studies, so (5) doesn't appear to be an accurate inclusion criteria.<br>Answer:<br>Corrected.<br>We have included interventional studies like single group pretest and post test studies , two group controlled studies(randomized& non randomized). If it is single grouped, we have taken only those studies with pretest and post test); If it is two group studies, didactic teaching as control. 5 points in eligibility criteria is based on PICOS framework.<br><br>3. Acronyms - Spell out all acronyms for first use. For all tables, please include the abbreviations and acronym definitions as a key.<br>Corrected<br><br>4. Page 13 - "face-to-face" and "face to face" (be consistent)<br>Corrected<br><br>5. Quality Assessment - A significant majority of studies used prior year scores as control with few studies attempting to control for confounding. Is it appropriate to pool the results of analyses?<br>We have separately mentioned pooled effect size of studies with historical group as control and studies with RCT design. Based on previous literature, and Cochrane guideline, we understand nothing wrong to pool this way. We pooled both conditions separately to avoid or adjust this dissimilarity or heterogeneity in the form of a subgroup analysis. Also, we presented the result as an overall to know the effectiveness blended learning as whole. As per Cochrane recommendations, all eligible studies can be included in the meta-analysis, regardless of the risk of bias assessment. Indeed, since almost all studies have low score, Cochrane suggests to present an estimated intervention effect based on all available studies, together with a description of the risk of bias in individual domains.<br>Reference: (Higgins JPT, Green S. Cochrane Handbook for systematic reviews of interventions version 5.1.0.: the Cochrane collaboration 2011)<br>As per the Cochrane Guideline of systematic review (section 9.6), we need to perform a subgroup analysis by splitting the data according to some specific characters such as participant, intervention or publication characters etc., to explore the effect of that particular factor in the analysis.<br>As its educational intervention study in pharmacy education, practically it is difficult to conduct prospective controlled trial in same batch of students in same academic institution. That's why most of the studies adopted historical group as control. Only three studies are RCTs. Out of 26 studies, we included 20 studies with no missing data for quantitative analysis. Among them 8 studies controlled confounding variables (please see quality assessment of studies by modified Ottawa scale). However, modified Ottawa scale requires controlling for subject characteristics by statistical covariate analysis. We have assigned "0" score for studies that tried to compare the baseline characteristics (adjusting confounders) by any method other than statistical covariate analysis. In all those studies, there were no statistically significant differences in students demographics / pre-test (Grade Point Average) between groups by t-test also.<br><br>6. Unequal weighted test scores - It appears by focusing on the mean difference, you equally weight 1 point on a 100 point exam vs. a 50 point exam, despite the point value being worth twice as much on the latter.<br>As per the Cochrane guideline, When studies have used different instruments to measure the same construct, a standardized means difference (SMD) may be used in meta-analysis for combining continuous data, hence we used SMD to combine our results. Reference link is provided.<br>https://handbook-5-1.cochrane.org/chapter_9/9_2_3_2_the_standardized_mean_difference.htm<br><br>Reviewer #2:<br>This study was a SR and MA that sought to assess the effectiveness of blended learning teaching strategies compared to traditional lectures. The study was well done. Methods were clear. Technical writing and grammar need to be revised. Attached is a

	file with grammatical revisions<br>Grammatical errors corrected.
Additional Information:	
Question	Response
**Financial Disclosure**<br><br>Enter a financial disclosure statement that describes the sources of funding for the work included in this submission. Review the submission guidelines for detailed requirements. View published research articles from *PLOS ONE* for specific examples.<br><br>This statement is required for submission and **will appear in the published article** if the submission is accepted. Please make sure it is accurate.<br><br>**Unfunded studies**<br>Enter: *The author(s) received no specific funding for this work.*<br><br>**Funded studies**<br>Enter a statement with the following details:<br>• Initials of the authors who received each award<br>• Grant numbers awarded to each author<br>• The full name of each funder<br>• URL of each funder website<br>• Did the sponsors or funders play any role in the study design, data collection and analysis, decision to publish, or preparation of the manuscript?<br>• **NO** - Include this sentence at the end of your statement: *The funders had no role in study design, data collection and analysis, decision to publish, or preparation of the manuscript.*<br>• **YES** - Specify the role(s) played.<br><br>* typeset	No fund received
**Competing Interests**<br><br>Use the instructions below to enter a competing interest statement for this submission. On behalf of all authors, disclose any competing interests that could be perceived to bias this work—acknowledging all financial support and any other relevant financial or non-	The authors have declared that no competing interests exist.

financial competing interests.<br><br>This statement **will appear in the published article** if the submission is accepted. Please make sure it is accurate. View published research articles from *PLOS ONE* for specific examples.<br><br>**NO authors have competing interests**<br><br>Enter: *The authors have declared that no competing interests exist.*<br><br>**Authors with competing interests**<br><br>Enter competing interest details beginning with this statement:<br><br>*I have read the journal's policy and the authors of this manuscript have the following competing interests: [insert competing interests here]*<br><br>* typeset	
**Ethics Statement**<br><br>Enter an ethics statement for this submission. This statement is required if the study involved:<br><br>• Human participants<br>• Human specimens or tissue<br>• Vertebrate animals or cephalopods<br>• Vertebrate embryos or tissues<br>• Field research<br><br>Write "N/A" if the submission does not require an ethics statement.<br><br>General guidance is provided below. Consult the submission guidelines for detailed instructions. **Make sure that all information entered here is included in the Methods section of the manuscript.**	As it is a systematic review, no ethics committee approval needed. review follows PRISMA guidelines.

Powered by Editorial Manager® and ProduXion Manager® from Aries Systems Corporation

**Format for specific study types**<br><br>**Human Subject Research (involving human participants and/or tissue)**<br>• Give the name of the institutional review board or ethics committee that approved the study<br>• Include the approval number and/or a statement indicating approval of this research<br>• Indicate the form of consent obtained (written/oral) or the reason that consent was not obtained (e.g. the data were analyzed anonymously)<br><br>**Animal Research (involving vertebrate animals, embryos or tissues)**<br>• Provide the name of the Institutional Animal Care and Use Committee (IACUC) or other relevant ethics board that reviewed the study protocol, and indicate whether they approved this research or granted a formal waiver of ethical approval<br>• Include an approval number if one was obtained<br>• If the study involved *non-human primates*, add *additional details* about animal welfare and steps taken to ameliorate suffering<br>• If anesthesia, euthanasia, or any kind of animal sacrifice is part of the study, include briefly which substances and/or methods were applied<br><br>**Field Research**<br><br>Include the following details if this study involves the collection of plant, animal, or other materials from a natural setting:<br>• Field permit number<br>• Name of the institution or relevant body that granted permission	
**Data Availability**<br><br>Authors are required to make all data underlying the findings described fully available, without restriction, and from the time of publication. PLOS allows rare exceptions to address legal and ethical concerns. See the PLOS Data Policy and FAQ for detailed information.	Yes - all data are fully available without restriction

A Data Availability Statement describing where the data can be found is required at submission. Your answers to this question constitute the Data Availability Statement and **will be published in the article**, if accepted.<br><br>**Important:** Stating 'data available on request from the author' is not sufficient. If your data are only available upon request, select 'No' for the first question and explain your exceptional situation in the text box.<br><br>Do the authors confirm that all data underlying the findings described in their manuscript are fully available without restriction?	
**Describe where the data may be found in full sentences. If you are copying our sample text, replace any instances of XXX with the appropriate details.**<br><br>• If the data are **held or will be held in a public repository**, include URLs, accession numbers or DOIs. If this information will only be available after acceptance, indicate this by ticking the box below. For example: *All XXX files are available from the XXX database (accession number(s) XXX, XXX.)*.<br>• If the data are all contained **within the manuscript and/or Supporting Information files**, enter the following: *All relevant data are within the manuscript and its Supporting Information files.*<br>• If neither of these applies but you are able to provide **details of access elsewhere**, with or without limitations, please do so. For example:<br><br>*Data cannot be shared publicly because of [XXX]. Data are available from the XXX Institutional Data Access / Ethics Committee (contact via XXX) for researchers who meet the criteria for access to confidential data.*<br><br>*The data underlying the results presented in the study are available from (include the name of the third party*	All relevant data are within the manuscript and its Supporting Information files

and contact information or URL).<br>• This text is appropriate if the data are owned by a third party and authors do not have permission to share the data.<br><br>* typeset	
Additional data availability information:	

Powered by Editorial Manager® and ProduXion Manager® from Aries Systems Corporation

Manuscript

Click here to access/download;Manuscript;Article page without track change.docx

TITLE PAGE

Type of paper: Systematic review and meta-analysis

Title of article: Effectiveness of blended learning in pharmacy education: A systematic review and meta-analysis

Running title: Blended learning in Pharmacy education

Authors name & Affiliation:

Athira Balakrishnan[a], Sandra Puthean[b], Gautam Satheesh[b,] M, K Unnikrishnan[b], Muhammed Rashid[a], Sreedharan Nair[a], Girish Thunga[a]

[a]Department of Pharmacy Practice, Manipal College of Pharmaceutical Sciences, Manipal Academy of Higher Education, Manipal, Udupi,Karnataka,India

[b]Department of Pharmacy Practice, National College of Pharmacy, Kozhikode, Kerala, India

Corresponding Author Details:

Dr. Girish Thunga, Assistant Professor, Department of Pharmacy Practice, Manipal College of Pharmaceutical Sciences, Manipal Academy of Higher Education, Manipal, Karnataka-576104, India

Phone number: +91-9880151127; Fax: +91-8202571998

E-mail address: girish.thunga@manipal.edu; ORCID ID: https://orcid.org/0000-0003-1257-969X

EFFECTIVENESS OF BLENDED LEARNING IN PHARMACY EDUCATION: A SYSTEMATIC REVIEW AND META-ANALYSIS

Abstract

Background & Objective: Though blended learning (BL), is widely adopted in higher education, evaluating effectiveness of BL is difficult because the components of BL can be extremely heterogeneous. Purpose of this study was to evaluate the effectiveness of BL in improving knowledge and skill in pharmacy education.

Methods:. PubMed/MEDLINE, Scopus and the Cochrane Library were searched to identify published literature. The retrieved studies from databases were screened for its title and abstracts followed by the full-text in accordance with the pre-defined inclusion and exclusion criteria. Methodological quality was appraised by modified Ottawa scale. Random effect model used for statistical modelling.

Key findings: A total of 26 studies were included for systematic review. Out of which 20 studies with 4525 participants for meta-analysis which employed traditional teaching in control group. Results showed a statistically significant positive effect size on knowledge (standardized mean difference [SMD]: 1.35, 95% confidence interval [CI]: 0.91 to 1.78, $p<0.00001$) and skill (SMD: 0.68; 95% CI: 0.19 to 1.16; $p=0.006$) using a random effect model. Subgroup analysis of cohort studies showed, studies from developed countries had a larger effect size (SMD: 1.54, 95% CI: 1.01 to 2.06), than studies from developing countries(SMD: 0.44, 95% CI: 0.23 to 0.65, studies with MCQ pattern as outcome assessment had larger effect size (SMD: 2.81, 95% CI: 1.76 to 3.85) than non-MCQs (SMD 0.53, 95% CI 0.33 to 0.74), and BL with case studies (SMD 2.72, 95% CI 1.86-3.59) showed better effect size than non-case-based studies (SMD: 0.22, CI: 0.02 to 0.41).

Conclusion: BL is associated with better academic performance and achievement than didactic teaching in pharmacy education.

Keywords: blended learning; flipped learning; education; knowledge; pharmacy; systematic review; teaching

Introduction

Evaluating the effectiveness of blended learning(BL), a thoughtful combination of both online and face-to-face instructions, is difficult because the components of BL can be extremely heterogeneous.[1,2] For instance previous systematic reviews / meta-analyses on BL have included multiple techniques such as virtual face-to-face interaction, simulations, online instruction, e-mails, computer laboratories, mapping and scaffolding tools, computer clusters, interactive presentations, handwriting capture, class room web sites, and virtual apparatuses.[3] Also, there is no standardized proportion in which BL combines online with face-to-face instructions.[4]

Flipped learning 'and 'hybrid learning' are often used interchangeably with BL. In flipped learning, the learner is first exposed to online content, which will be reinforced during face-to-face sessions. [5] Hybrid learning, a combination of face-to-face instruction with computer mediated instruction, is most often used in United States. [6] In all forms of BL, the learner enjoys a certain degree of autonomy in deciding the pace of learning. However, previous reported systematic reviews on BL have not taken the keyword "flipped" in their search strategy [7,8]

Increased research has been published on BL in medical education over last decades. For instance, Quian Liu et al's systematic review and meta-analysis reported that BL has consistent positive effects in comparison with no intervention for knowledge acquisition in health

profession [7]. In another systematic review, McCutcheon et al reported a deficit of evidence on implementation of BL in undergraduate nursing education. [9] Most of the published systematic review and meta-analyses in medical education were focused on medical students or nursing students or other healthcare professionals [8,910] There is only one meta-analysis that evaluates the effectiveness of flipped learning in pharmacy education, with a major limitation namely, lack of prospective randomized control trials (RCT) and restrictions to the domain of flipped contexts. [11] Accordingly, we designed our objective to assess the effectiveness of BL which employed a combination of online and face-to-face instruction in blended, hybrid and flipped contexts in pharmacy education. We have considered BL as a combination of online and face-to-face instruction, excluding other computer mediated forms like virtual labs, gamifications, simulations to limit heterogeneity and included all possible synonyms of blended, hybrid, flipped learning and pharmacy education.

Materials and Methods

This study followed Preferred Reporting Items for Systematic Reviews and Meta-Analyses (PRISMA) Guidelines (PRISMA Checklist attached in data appendix S1)

Eligibility Criteria

We employed PICOS (population, intervention, comparison, outcome, and study design) framework for the inclusion of studies. Studies were considered eligible, if they: (1) were conducted among pharmacy students, (2) used a BL intervention in the experimental group, (3) used traditional lecture based learning as control for two arm studies and pre-test score for single arm studies (4) reported knowledge score/ objective structured clinical examination (OSCE) score as outcome (5) were two groups controlled studies (randomised/non-randomised)/ single group pre-test-post- test studies.

We excluded studies which did not explicitly state components of BL i.e. face-to-face learning and computer assisted learning. Computer assisted learning can be any form of technologies like online learning, e-learning, video podcasts, or the application of university learner management system for posting lectures. We excluded studies which employed “virtual face-to-face” interactions (as practiced by universities with satellite campuses). Studies which did not report quantitative outcome of knowledge (comparison of students who completed and did not complete online module, number of correct answers between the groups, comparison of pass percentage), studies which evaluated only online component of BL, and surveys were also excluded. Multi-year studies without differentiating between study term years were excluded. Reviews, short communication, conference proceedings, editorials, meeting abstracts and non-English studies were also excluded.

Data Sources and literature search:

A literature search employing PubMed, Scopus and Cochrane Library, was performed using a comprehensive search strategy since the inception of each database up to mid-December 2020. We employed all the MesH terms and key words for "BL" (Blended learning, blended course, blended program, hybrid learning, hybrid Course, Hybrid Program, Hybrid training, Flipped learning, Flipped Course, Flipped Program, Computer-aided learning, Computer-assisted learning, Integrated learning, Distributed learning, Distributed education Integrated instruction, Computer-aided instruction, Computer-assisted instruction) and “Pharmacy Student" which was obtained from the databases and previous studies. We employed the asterisk (*) as a wildcard character in keyword searches. We also searched for additional reference materials by consulting the cross references listed in the included publications, in addition to Google and Google Scholar. (Details in data appendix S2)

Study selection and Data Extraction

The retrieved studies from databases were screened for its title and abstracts followed by the full-text in accordance with the pre-defined inclusion and exclusion criteria. (List of excluded studies provided in Data appendix S3). We compiled and collated data in a comprehensive data extraction form containing characteristics such as, author and year of publication, population, duration and subject covered, nature of BL, sample size, outcomes. The above data extraction form was perfected by trial and error, by piloting on 3 articles. Three independent reviewers were involved in study selection and data extraction to limit the bias and any disagreements were resolved through consensus or by discussion with another member of research team.

Quality Assessment

Modified Newcastle Ottawa scale (Newcastle Ottawa scale-education) was used to appraise methodological quality of included studies. [12,13,14] This tool assesses the following criteria: 1) representativeness of intervention group (1 point) 2) selection of comparison group (1point) 3) comparability of comparison group (2 point) 4) study retention (1 point) 5) blinding of assessment (1 point), totalling a maximum of 6 points. Two independent reviewers were involved to appraise the methodological quality to limit the bias and any disagreements were resolved through consensus or by discussion with another member of research team.

Data Synthesis

The evidence were synthesised narratively and presented in tabular form. We employed meta-analysis whenever possible. We omitted studies from data pooling whenever data did not meet the requirements of meta-analysis, such as, participant number, mean and standard deviation [SD]. All comparisons were based on scores of consecutive years. If more than one topic was delivered by BL in same study with separate scores for each, we considered them as separate studies. RevMan 5.3 was used to conduct the meta-analysis.[15] The data were used as mean with SD and outcomes were presented as standardised mean difference (SMD) along with 95%

confidence interval (CI). Studies that did not report a SD, the corresponding SD from the p-values and standard errors were generated as per Cochrane guideline [16]. Heterogeneity was assessed by I^2 statistics and random effect model used for statistical modelling. Subgroup analysis were performed to find out potential source of heterogeneity based on factors like studies with case studies and without case studies, studies which reported outcome as a measure of multiple choice questions(MCQs) or non MCQs, and studies from developed and developing countries. Sensitivity analysis were performed to ensure the robustness of findings.

Publication bias

We employed a funnel plot for visual inspection of publication bias, which was assessed for statistical significance by Egger's and Begg's test.[16]

Results

A total of 2539 records were retrieved first, of which 2448 underwent initial screening. Next, 2383 studies were omitted, yielding 65 full-text studies, of which 26 studies were included for systematic review, and 20 for meta-analysis. (See figure 1 for details of study selection)

Characteristics of studies included for systematic review

Of the 26 studies included, only two employed single arm pre-test-post-test design.[17,18] The remaining 24 studies were controlled studies[19-42] out of which 19 used examination scores of previous year[19-34, 36-41] and one used examination score of subsequent year as control.[35] There were 3 randomised trials[19,14,31] out of which one was cluster randomised.[24] Another study divided learning materials into didactic and BL in same population.[28] 18 studies originated from USA and 8 studies from other countries.[17,20-21,23-25,26,28,33] (See table 1 for characteristics of included studies)

Table 1: Characteristics of included studies

Author	Country	Population	Topic(duration)	Intrv. details	Study design	Sample size		Post-intrv. academic outcome	Other activities	Mean Result Score		Major outcome
						Intrv.	***Control***			***Intrv.***	***Control***	
Wilson et al [40] (2019)	USA	2nd year pharmaco-therapy students	Selected self-care pharmaco-therapy(NC)	Online (Vimeo) + class activities	Cohort (compared with previous year students' score)	N/A	N/A	Exam performance (65% course grade)- Assessment questions	TBL	83.5%	83.3%	No statistically significant differences in student outcomes
Newsom et al[34] (2019)	USA	1st year students enrolled in spring 2015-17. (Control: spring '14)	Pharmaco kinetics(NC)	Traditional class and video podcast	Cohort Intrv.: 2015-17 Didactic teaching: 2014	2015: 153 2016: 152 2017: 153	2014: 175	Final exam score: questions based on Bloom's taxonomy.	Case based practice problems	2015: 85.8(7.7) 2016: 85.1(9.2) 2017: 78(12)	2014: 77.6 (13.3)	Final exam scores were significantly higher in spring '15 and '16 compared to '14 ($p<0.001$). 2017 scores were similar to that of 2014.
GoH et al[23] (2019)	Malaysia	2nd year Dosage Form II course	3 credit course. Dosage form II(NC).	Pre-recorded video + F2F sessions	Cohort: Two group comparison ('16 & '17 batch)	63	74	Final Exam score. Subjective (5 MCQ + 2 essay)	Online games	49.93	41.24	Final exam performance significantly higher in the flipped classroom group
He et al[24] (2019)	China	Junior year pharmacy under-graduates	Pharma-ceutical marketing (4 months)	Online + class	Cluster randomization	81	56	Final score (subjective – short answer, essays, MCQ)	Case discussion Group activity	88.21 ± 5.95	80.05 ± 5.59	Compared with LBL methods, implementing the FC model improved student performance.
Kouti et al[28] (2018)	Iran	Pharmacy Students (2015-16 batch)	Non-prescription drugs (1 semester)	Electronic based + lecture based	Propective comparative study – 3-group study (f2f, Electronic, BL)	57	-	Final exam score (not clear)	Case studies	E-learning group: 16.17 ± 0.33; Lecture group: 13.75 ±	-	BL method and an e-learning approach can positively influence students' knowledge towards non-prescription drugs

										0.16; BL: 16.39 ± 0.19		
Kangwantas et al[26] (2017)	Thailand	2nd year pharmacy students	Fundamental nutrition (1 year)	Videos (moodle platform) +class activities	Cohort compared with previous year score.	29	21	Pre and post-test within the group Post-test: main exam scores. (subjective-MCQ + short answer)	Case discussion	Pre-test: (7.45±1.89 and Post-test: (8.17±1.44) not statistically different (p =0.08).	Flipped class scored higher (7.24±1.24 vs. 6.19±1.76) (p =0.028)	Student performance as measured by final scores of the module was better than those for the same module taught with a traditional lecture in previous year
Koo et al[27] (2016)	USA	2nd year PharmD students	Pharmaco-therapy (1 year)	Online + F2F discussion	Cohort-comparison (2011 & 2012)	89	89	Exam score: Objective, MCQ	Case study discussion	88.2% (7.3%)	83.4% (7.9%)	The redesigned course improved student test performance and perceptions of learning experience
Giuliano et al[22] (2016)	USA	1st year Pharmacy Students	Drug literature evaluation (1 year)	Youtube lecture + Class session.	Cohort: 2-group study (2013 & 2014)	94	99	Final exam score: subjective-application, analysis & Bloom's taxonomy evaluation	Group activities	86.1%	75.6%	The flipped model is an excellent fit for drug literature content and courses that want to incorporate more active learning
Edginton et al[21] (2013)	Canada	2nd year pharmacy students	Bio-chemistry (1 year)	online + classroom	Cohort: 2011 vs. 2010	116	109	Final grade: subjective-MCQ + calculation + long answer	Group discussion Case studies	78.8 + 11.7	61.8 + 17.8	The student driven BL model correlated positively with increased interest and perceived and actual learning gains. P<0.00001.
Pierce [36](2012)	USA	Pharmacy students	Renal pharmaco-therapy (8 weeks)	Video podcast + classroom	Pre-test and post-test-within the group (2012) Only post-test between	71	N/A	Objective-MCQ	Case discussion	Pre-test (33.5 ± 11.6 and post-test (79.2 ± 10.6): within the group	77.7 ± 4.7	Implementing a flipped classroom to teach renal pharmacotherapy resulted in improved student performance

					groups (2012 & 2011)					Between groups: 81.6 ±4.4		and favourable student perceptions
McLaughlin et al[31] (2015)	USA	PharmD students	Neuro-logic pharmaco-therapy(NC).	e-learning + class	Randomized (same class)	57	59	Final exam score: 9 final exam questions-not clear whether questions are MCQ/subjective.	Case studies	80.12 + 13.57	74.76 + 15.12	Interactive online preparatory tool improves student learning in neurologic pharmacotherapy.
Wong et al[41] (2014)	USA	1st year pharmacy students	Cardiac arrhythmia (3 classes)	Pre-recorded video + class	Cohort: compared with previous year (2012 & 2011)	101	103	Final exam score: 5-6 MCQ on cardiac arrhythmias.	Case based exercises	Basic science: 88.3±1.9 Pharmacology: 89.6±2 Therapeutics: 89.2±1.4	84.1 ± 1.9; 56.8 ± 2.2; 73.7 ± 2.1	Use of the flipped teaching in a 3-class pilot on cardiac arrhythmias improved exam scores for pharmacology and therapeutics classes.
Anderson et al[19] (2017)	USA	1st year pharmacy students	Pharma-ceutical calculations (6 weeks)	recorded lecture + video	Randomized	38	32	Final exam-Skill: OSCE Score at 6 weeks	Case studies	71.3 (14.7)%	61.8 (17.7)%	Average OSCE performance was be higher in flipped model than lecture model
Cotta et al[20] (2016)	Georgia	1st year pharmacy students	Pharma-ceutical calculation (10 weeks)	Pre-recorded video + class	Cohort: 2012 vs. 2011	151	165	Final exam part 2 score-objective graded quizzes	-	88.3 (9.5)	84.1 (11.3)	Flipped classroom can improve student performance and satisfaction in pharmaceutical calculations (P<0.001)
Lancaster[29] (2011)	USA	2nd PharmD	OTC, medicines (15 weeks)	Pre-recorded video + class	Cohort: 2008 vs. 2009	97	97	Final Exam scores-objective-9 quizzes	Clinical based cases with Group discussion Puzzles,	84.09	65.15	Students performed significantly higher on quizzes and examinations when using this hybrid teaching model.

									Think pair share activities.			
Stewart[38] (2013)	USA	3rd year pharmaco-therapy	Pharmaco - therapy(NC)	Podcast + active learning	Cohort: 2009 vs. 2010	71	65	Final exam score: 20 MCQ	Group discussion	72.9+1.5 (12.63)	77.15+1 .2 (9.6)	The class averages on the final exams were significantly higher for 2009 compared to 2010 batch (P: 0.019).
Lockman et al[30] (2017)	USA	1st year pharmaco-logy & therapeutic s course	Pain manag-ement module(NC)	E learning + in class lecture	Cohort: 2015 vs. 2016	162	156	OSCE: skill Knowledge-MCQ	Cases & mini cases, quiz games, Mind-mapping debates	MCQ: 82.30% (SD 10.25) OSCE: 79.34(9)	77.23% (SD 12.43 OSCE: 67.01(9. 6)	Student performance improved significantly after flipping the content of pain management module.
Nazar et al[33] (2018)	Qatar	Stage 2 Pharmacy under-graduate students	Pharmacy law(NC)	Online class + In-class activities.	Cohort study: compared with previous year	69 (2016-17)	63 (2015-16) 37 (2014-15)	Final summative examination score	Group discussion	82.2 (6.3)%	84.2 (6.8)% 83.0 (7.6)%	Examination performance appeared to be unaffected by the change in teaching style
Hughes et al[25] (2016)	USA	P1 pharmacy students	Drug information (5 weeks)	Narrated video + F2F lab session	Cohort study: compared with previous years (2012 Vs. 2013)	127	121	Final exam score: Objective-40 MCQ	-	88.99%	84.87%	Mean final exam scores significantly increased ($p < 0.05$)
Gloudeman et al[42] (2017)	USA	1st year pharmacy students	Pharmaceutical calculation (6 week)	Online video + classroom session	Cohort study: compared with previous years (2015 Vs. 2014)	102	104	Final exam score: 13 pharma-ceutical calculation questions	-	80.5 ± 15.8%	77.8 ± 16.8%	The mean exam scores of the intrv. were not significantly different than the control (p= 0.253)
Czepula et al[17] (2017)	Brazil	Under-graduate bachelor's	Pharma-ceutical care(NC)	F2F + distance learning	Quasi-experimental, prospective.	Pharma-ceutical care 1: 82		Pre- and post-test: 30 MCQ	-	Module 1: Mean scores increased		Positive results were observed regarding the students'

		degree in pharmacy			Two groups. Both groups received BL of pharma-ceutical care 1 & 2.	Pharmaceutical care 2: 51				from 4.8 to 6.3 (p<0.05) Module 2: Mean scores increased from 4.1 to 5.5 (p<0.05)		performance in the two disciplines
Prescott et al[37] (2016)	USA	1st and 2nd year pharmacy students	Two course: PA1 and PA2(NC)	Online videos + class	Cohort study: Comparison of traditional and BL of PA1 & PA2 (2014-15 vs, 2013-14)	PA1: 130 PA2: 131	PA1: 126 PA2: 122	Final examination score: 20 short answer questions	Group discussion (TBL) Case based learning	Knowledge: PA1: 80.5 (9.6) PA2: 80.6 (14.3) P<0.001 Skill: PA1: 93.1 (7.6) PA2: 83.5 (12.5)	Know-ledge: PA1: 73(12); PA2: 74.5(12.1) P<0.001 Skill: PA1: 89.1 (13.8) PA2: 81.5 (12.6)	BL was associated with improved academic performance and was received by students.
Wanat et al[39] (2016)	USA	3rd year pharmacy students	. Critical care 2hr credit course(4 weeks)	Video recorded lecture + in-class activities	Cohort study: Compared with previous year score. (2013, 2014 Vs. 2012, 2011)	51	54	Overall exam performance: subjective: online quiz + skills: examining patients	Group learning	87.7% (3.7)	82.6% (6.3)	Exam scores of students in BL group is significantly higher than control
Phillips et al[35] (2016)	USA	1st and 2nd year PharmD students	EBM & Therapeutics(6 month)	Online video prior to class room	Cohort study: Knowledge compared with previous year scores - two group comparison.	EBM: 201 Therapeutics: 199	N/A	Final Exam score/-not clear whether questions are MCQ/subjective.		EBM:83% Therapeutics : 97	EBM:85% Therapeutics: 98%	Use of the BLE did not seem to have an impact on long-term knowledge in this study

Hess et al[18] (2016)	USA	2nd year pharmacy students	Patient centred communic ation skills(One semester,)	Online modules + small group discussion	Single group study	57	-	Pre-test and post-test<br><br>OSCE	Group discussion	Significant Increase in scores from pre-test to post-test.7 domains of pre and posttest scores provided.		Patient-centred interprofessional communication skills improved significantly with BL
McLaughlin et al[32] (2014)	USA	1st year pharmaceut ics students	Pharmace utics course(N C)	Flipped classroom (iLAMs + F2F)	Cohort study: Traditional vs. Flipped 2012 vs. 2011	162	153	Final exam grade. Subjective-quizes+exami nation scores	-	Final score; 165.48 ± 13.34	160.06 ± 14.65	Higher final exam grades in flipped classroom

Intrv.: Intervention; BL: Blended learning, EBM: Evidence-based medicine; TBL: Team Based Learning; F2F: Face-to-Face, N/A-not Available, NC-not clear MCQ: Multiple choice questions, OSCE: Objective structured clinical examination, PA1: Patient assessment 1 course, PA2: Patient assessment 2 course, iLAMS.: integrated learning accelerator module

Outcome measured

Only 3 studies[18,19,39] reported outcome as skills(patient centred interpersonal communication skills, students' performance on pharmaceutical calculation, and critical care therapeutics) while 21 studies reported only knowledge score. [17, 20-29, 31-36, 38, 40-42] Two reported both knowledge and skills as outcomes.[30, 37] Outcomes were measured variably as mean examination percentage (n=16) or mean examination score (n=6) or objective structured clinical examination (OSCE) (n=2). Two studies reported both examination percentage and OSCE score.

BL approaches

Two studies employed face-to-face session followed by online activities [17, 34] while all other studies employed face-to-face session after watching online content. Only one study reported time spent and workload associated with BL.[37]

Quality assessment of included studies

As per modified Ottawa scale requirements, we ascertained that intervention groups in all the included studies were representatives of target population. Out of 26 studies, 19 used previous year students' score as control, one used subsequent year score as control and 3 studies were randomized. Two studies used analysis of covariance(ANCOVA) for controlling covariates in final analysis [23 ,38] and one used linear regression.[22] In five studies there were no statistically significant differences in students demographics / pre-test (Grade Point Average) between groups by t-test. [27, 30, 32, 34, 41] However, modified Ottawa scale requires controlling for subject characteristics by statistical covariate analysis. Outcome assessment was blinded for 11 studies, as assessor cannot be influenced by group assessment (third party statistician) or assessments did not require human judgments (MCQs/ graded performance). [17,

[19-20, 25, 27, 29-30, 36, 38, 40-41] As all studies were part of curriculum in educational institution, there is no mention about drop outs. All studies obtained a score below 4 except one.[19] (See data appendix S4)

Quantitative analysis

We included 20 studies with 4525 participants for meta-analysis that employed traditional teaching in the control group and had no missing data.

Efficacy of BL versus. Traditional teaching in improving knowledge

Pooled effect of 18 studies showed that knowledge improved significantly in BL, with large effect compared to didactic teaching ((SMD 1.35, 95% CI-0.91 to 1.78, $p<0.00001$). In the knowledge domain, randomised controlled studies had a lower pooled effect (SMD 0.88) than cohort studies (SMD 1.41). There was significant statistical heterogeneity among studies ($I^2=98\%$, $p<0.00001$) with individual effect sizes ranging from −0.37 to 15.54. (See Figure 2)

Efficacy of BL versus traditional teaching in improving skill

Pooled effect size (SMD 0.68, 95% CI: 0.19 to 1.16,Z=2.74,p=0.006) of 4 studies in improving skills, showed statistically significant moderate to large effect, compared with didactic teaching. Significant statistical heterogeneity was observed among studies ($I^2=92\%$, $p<0.00001$). (See figure 3)

Subgroup Analysis

Subgroup analysis of cohort studies, in the knowledge domain, demonstrated advantage for BL over traditional teaching, in developed countries (SMD 1.54, 95% CI 1.01- 2.06) than developing countries (SMD 0.44, 95% CI 0.23-0.65). Studies which employed MCQ scores as outcome showed larger effect size (SMD 2.81, 95% CI 1.76-3.85) than non MCQs (SMD 0.53,

95% CI 0.33 -0.74). Also, studies which employed case studies/case discussion favoured BL (SMD 2.72, 95% CI 1.86-3.59) than non-case based studies (SMD: 0.22, CI: 0.02 to 0.41). Subgroup analyses of studies improving skill were not performed, as all studies originated from United States of America and all employed case studies/case discussion. (See table 2)

Table 2: Subgroup Analysis of Cohort studies

Study Characteristics:	Sample size	Test for heterogeneity			Test for effect	
		I^2(%)	Q statistics	P value	Pooled effect size(SMD(C1))	P value
1. Country						
Developed	3731	98	854.67	P<0.00001	1.54(1.01,2.06)	P<0.00001
Developing	366	0	0.89	P=0.35	0.44(0.23,0.65)	P<0.0001
Total	4097	98	857.3	P<0.00001	1.41(0.94,1.87)	P<0.00001
2. Outcome assessment						
MCQ	2002	99	796.46	P<0.00001	2.81(1.76,3.85)	P<0.0001
Non MCQ	1635	76	29.47	P<0.0001	0.53(0.33,0.74	P<0.0001
Not clear	460	96	24.89	P<0.00001	0.23(-0.80,1.25)	0.66
Total	4097	98	857.35	P<0.00001	1.41(0.94,1.87)	P<0.00001
3. Case studies						
Present	2364	99	736.66	P<0.00001	2.72(1.86,3.59)	P<0.00001
Absent	1733	75	31.53	P<0.0001	0.22(0.02,0.41)	0.03

Total	4097	98	857.36	P<0.00001	1.41(0.94,1.87)	P<0.00001

MCQ: Multiple choice questions; SMD: Standardised mean difference; CI: confidence interval

Sensitivity Analysis

A sensitivity analysis was performed in studies improving knowledge by removing two studies (Wong et al., [2&3]) which are having lesser weight (3.1% and 4.1%, respectively), and higher outlier (MD: 15.54 and 8.64, respectively) which supported the main results (SMD: 0.55; 95%CI: 0.33 to 0.77). The result of sensitivity analysis is depicted in Figure 4.

Publication bias

Visual inspection of funnel plot revealed an obvious asymmetry, demonstrating possible publication bias. This was confirmed by Egger's (P=0.00006) and Begg's (P=0.04) test. (See figure 5)

Discussion

This systematic review and meta-analysis primarily attempted to evaluate the impact of BL approach on various outcomes in pharmacy education. We identified 26 studies relevant for systematic review, in which 18 demonstrated significant improvement in learning outcome, against controls. Two of them were single arm studies which also showed improved performance after intervention. 24 of the 26 studies included in this systematic review were controlled, among which majority (n=19) employed examination scores of previous year(s) as the control. All studies employed first online review of contents followed by face-to-face discussion except two. Studies which employed face-to-face discussion followed by online activities also favoured BL. [17, 34] Face- to- face discussion part of BL in all included studies

involved either reinforcing the concepts by tutor or using learning strategies such as case studies, case discussion or group activities.

In addition to the general scarcity of literature comparing BL and traditional methods, a major limitation of the previous meta-analysis by Gillette et al., was the lack of prospective RCTs. [11] Our meta- analysis included 20 of the studies included in the systematic review. Our review included 3 RCTs, all of which showed major improvements in either knowledge score or skill. We report a large pooled effect size for knowledge and a medium to large for skills. These findings were statistically significant with high heterogeneity in all analyses and are consistent with those reported by previous meta-analyses in medical education.

Majority of the studies reported knowledge score in terms of either mean examination percentage/score or OSCE, whereas 5 studies reported outcomes based on skill. Many of the studies included in this review also reports that BL has a major effect on improving teaching as well as positive student perceptions about learning. As mentioned earlier, the rich variety of components can attribute to enhanced learning experience as well as increased engagement and learning activities such as group assessment, assessment quizzes and peer discussions. Even the studies that did not report significant difference in acquisition of knowledge– such as those by Phillips et al., and Gloudeman et al. showed that the perceptions of both students and faculty favoured BL. [35, 42]

Another important finding is that BL modules which employed case studies/discussions or case-based scenarios reported better outcomes. A few studies also concluded that positive results obtained may not be attributed entirely to the suggesting that case studies need to be included in learning strategies. [24, 37] There is evidence to show that case studies simulate real world situations and enhance interactive student-centred learning, particularly in the health professions. Incorporating case studies in a real-world context is extensively useful in pharmacy education, as it enhances students' complex decision-making abilities.

Out of 26 studies, only 4 originated from developing countries, possibly because of poor online connectivity, lack of resources, fear of adopting unfamiliar technology, lack of skill development program to instructors, interruption in power supply and internet connections, affordability, low bandwidth and trust deficit. **[20, 17, 26, 28]**. A single study that compares time budgets reported that BL techniques were completed ahead of allotted time.**[35]** BL approach appears to significantly improve the learning outcomes in pharmacy students and reason could be following,

(i) **Relaxed/flexible scheduling**: BL allows students to view electronic materials at their own pace and time

(ii) **Improved interaction**: BL makes classroom discussion more meaningful because of content familiarity.

(iii) **Variety of components**: BL incorporates a rich variety of face-to-face and online components.

This study has few limitations. First, the search was restricted to the publications in English language, which might have contributed to missing out eligible studies in non-English speaking countries. However, a comprehensive search in various databases would have covered the maximum quality publications. Second, our review also excludes conference proceeding and unpublished or grey literature. However, this may increase the credibility of our findings obtained from full length papers by avoiding the irrelevant or incomplete acquisition of the data. Third, there was high heterogeneity among the outcomes or measures of outcome, thereby restricting our choice exclusively to studies reporting quantitative outcomes. Fourth, the heterogeneous administration pattern of BL was an another challenge in this review, so we included those studies which used online teaching along with face-to-face approach, this made our result more robust and conclusive. Statistical heterogeneity was high in all analysis. However, this is in accordance with other meta-analysis in medical education [7,8,43] Subgroup

analyses did not find any source of heterogeneity. Despite the effective search strategy, one major limitation is the majority i.e. 18 of the 26 studies, were from the US, which could impact the global representativeness of the findings. Therefore, future research should address the impact of BL in diverse populations from other countries.

Publication bias was addressed by including the three major scientific databases (Pubmed, SCOPUS and Cochrane) during the literature search. This resulted in increased number of papers which may have further increased the likelihood of selecting papers with negative results. In our review, 5 of the 26 studies reported that BL yields either equal or poorer outcomes than didactic teaching.[42, 33, 35, 38, 40]

Conclusion

BL is associated with better academic performance and achievement than didactic teaching in pharmacy education. The COVID-19 pandemic is radically reshaping education sector to transform from conventional teaching to more online learning. In this scenario, it is critical _to conduct more controlled empirical studies to evaluate the effectiveness of BL. Such research can inform education policies and guidelines to standardise blended learning.

Declaration

Funding: No funding is received from any sources or funding agency for this study.

Conflicts of interest/Competing interests

Authors report no conflicts of interest to declare. The listed authors are solely responsible for the originality and content of the manuscript. The authors have no relevant affiliations or financial involvement with any organization or entity with a financial interest in or financial conflict with the subject matter or materials discussed in the manuscript. This includes

employment, consultancies, honoraria, stock ownership or options, expert testimony, grants or patents received or pending, or royalties.

Availability of data and material

The datasets generated during and/or analysed during the current study are not deposited in any repository and it is available from the corresponding author on reasonable request.

Ethics approval

An ethical approval was not required as the study was not included any patients.

References

1. Graham CR. Emerging practice and research in blended learning. In M. G. Moore (Ed.), Handbook of distance education. New York: Routledge.2013:333-350

2. Dziuban C *et al*. Blended learning: the new normal and emerging technologies. ETHE. 2018; 15(1): 3.

3. Means B *et al.* The effectiveness of online and blended learning: A meta-analysis of the empirical literature. *Teach. Coll. Rec.* 2013;115(3): 1-47.

4. Garnham C. Introduction to hybrid courses. Teaching Technology Today. 2002;8.

5. DeLozier SJ *et al.* Flipped classrooms: a review of key ideas and recommendations for practice. *Educ. Psychol. Rev.* 2017; 29(1):141-151.

6. O'Byrne WI *et al.* Hybrid and blended learning: Modifying pedagogy across path, pace, time, and place. *J Adolesc Adult Lit*. 2015;59(2):137-140.

7. Liu Q *et al.* The effectiveness of blended learning in health professions: systematic review and meta-analysis. *J Med Internet Res.* 2016;18(1): e2.

8. Vallée A, Blacher J, Cariou A, Sorbets E. Blended Learning Compared to Traditional Learning in Medical Education: Systematic Review and Meta-Analysis. *J Med Internet Res.* 2020;22(8):e16504

9. McCutcheon K *et al.* A systematic review evaluating the impact of online or blended learning vs. face-to-face learning of clinical skills in undergraduate nurse education. *J Adv Nurs.* 2015;71(2):255-270.

10. Rowe M, Frantz J, Bozalek V. The role of blended learning in the clinical education of healthcare students: a systematic review. *Medical teacher.* 2012;34(4):e216-21

11. Gillette C *et al.* A Meta-Analysis of Outcomes Comparing Flipped Classroom and Lecture. *Am J Pharm Educ.*2018; 82(5):6898.

12. Cook DA *et al.* Method and reporting quality in health professions education research: a systematic review. *Med Educ.* 2011;45(3):227-238.

13. Cook DA *et al.* Internet-based learning in the health professions: a meta-analysis. *JAMA.* 2008; 300(10):1181-1196.

14. Cook DA *et al.* Appraising the quality of medical education research methods: The Medical Education Research Study Quality Instrument and the Newcastle-Ottawa Scale-Education. *Acad Med.* 2015;90(8):1067-1076.

15. Collaboration, T. C. (2014). Review Manager. Copenhagen.

16. Higgins JP *et al.* Cochrane handbook for systematic reviews of interventions: John Wiley & Sons.2019.

17. dos Santos Czepula AI *et al.* Active methodology and blended learning: An experience in pharmaceutical care. *Curr Pharm Teach Learn.* 2018;10(1):106-111.

18. Hess R *et al.* Teaching communication skills to medical and pharmacy students through a blended learning course. *Am J Pharm Educ.* 2016;80(4).

19. Anderson HG *et al.* Comparison of pharmaceutical calculations learning outcomes achieved within a traditional lecture or flipped classroom andragogy. *Am J Pharm Educ.* 2017;81(4).

20. Cotta KI *et al.* Effectiveness of flipped classroom instructional model in teaching pharmaceutical calculations. *Curr Pharm Teach Learn.* 2016;8(5):646-653.

21. Edginton AN *et al.* Using student feedback to design a more effective clinical biochemistry course component. *Curr Pharm Teach Learn.* 2013;5(1):23-32.

22. Giuliano CA *et al*. Evaluation of a flipped drug literature evaluation course. *Am J Pharm Educ*. 2016;80(4).

23. Goh CF *et al.* Flipped classroom as an effective approach in enhancing student learning of a pharmacy course with a historically low student pass rate. *Curr Pharm Teach Learn.* 2019; 11(6):621-629.

24. He Y *et al*. The effects of flipped classrooms on undergraduate pharmaceutical marketing learning: A clustered randomized controlled study. *PLOS One*. 2019;14(4), e0214624.

25. Hughes, P. J., Waldrop, B., Chang, J. J. C. i. P. T., & Learning. (2016). Student perceptions of and performance in a blended foundational drug information course. *Curr Pharm Teach Learn.* 2016; 8(3):359-363.

26. Kangwantas K *et al*. Implementing a flipped classroom approach to a course module in fundamental nutrition for pharmacy students. *Pharm. Educ.* 2017; 17.

27. Koo CL *et al.* Impact of flipped classroom design on student performance and perceptions in a pharmacotherapy course. *Am J Pharm Educ*. 2016;80(2).

28. Kouti L *et al.* Comparison of the effectiveness of three educational methods (e-learning, lectures and blended) on pharmacy students' knowledge of non-prescription drugs. *Pharm. Educ.* 2018; 18:197-201.

29. Lancaster JW *et al.* Online lecture delivery paired with in class problem-based learning… Does it enhance student learning? *Curr Pharm Teach Learn.*2011;3(1):23-29.

30. Lockman K *et al.* Improved learning outcomes after flipping a therapeutics module: results of a controlled trial. *Acad Med.* 2017;92(12):1786-1793.

31. McLaughlin JE *et al*. Comparison of an interactive e-learning preparatory tool and a conventional downloadable handout used within a flipped neurologic pharmacotherapy lecture. *Curr Pharm Teach Learn.* 2015;7(1):12-19.

32. McLaughlin JE *et al.* The flipped classroom: a course redesign to foster learning and engagement in a health professions school. *Acad Med.* 2014;89(2):236-243.

33. Nazar H *et al.* A study to investigate the impact of a blended learning teaching approach to teach pharmacy law. *Int J Pharm Pract.* 2019;27(3):303-310.

34. Newsom L *et al.* Implementation and evaluation of problem-based video podcasts in an introductory pharmacokinetics course. *Curr Pharm Teach Learn.* 2019; 11(12):1213-1220.

35. Phillips JA *et al*. Time spent, workload, and student and faculty perceptions in a blended learning environment. *Am J Pharm Educ.* 2016;80(6).

36. Pierce R *et al*. Vodcasts and active-learning exercises in a “flipped classroom” model of a renal pharmacotherapy module. *Am J Pharm Educ.* 2012;76(10).

37. Prescott WA *et al.* Introduction and assessment of a blended-learning model to teach patient assessment in a doctor of pharmacy program. *Am J Pharm Educ.* 2016;80(10).

38. Stewart DW *et al.* An analysis of student performance with podcasting and active learning in a pharmacotherapy module. *Curr Pharm Teach Learn.* 2013;5(6):574-579.

39. Wanat MA *et al.* A critical care hybrid online elective course for third-year pharmacy students. *Am J Pharm Educ.*2016;80(9).

40. Wilson JA *et al.* Flipped classroom versus a didactic method with active learning in a modified team-based learning self-care pharmacotherapy course. *Curr Pharm Teach Learn.* 2019;11(12):1287-1295.

41. Wong TH et al. Pharmacy students’ performance and perceptions in a flipped teaching pilot on cardiac arrhythmias. *Am J Pharm Educ.* 2014;78(10).

42. Gloudeman MW *et al.* Use of condensed videos in a flipped classroom for pharmaceutical calculations: Student perceptions and academic performance. *Curr Pharm Teach Learn*. 2018;10(2):206-210.

43. Fontaine G, Cossette S, Maheu-Cadotte MA, Mailhot T, Deschênes MF, Mathieu-Dupuis G, Côté J, Gagnon MP, Dubé V. Efficacy of adaptive e-learning for health professionals and students: a systematic review and meta-analysis. *BMJ open*. 2019 ;9(8): e025252.

 Click here to access/download;Figure;Figure 1.docx

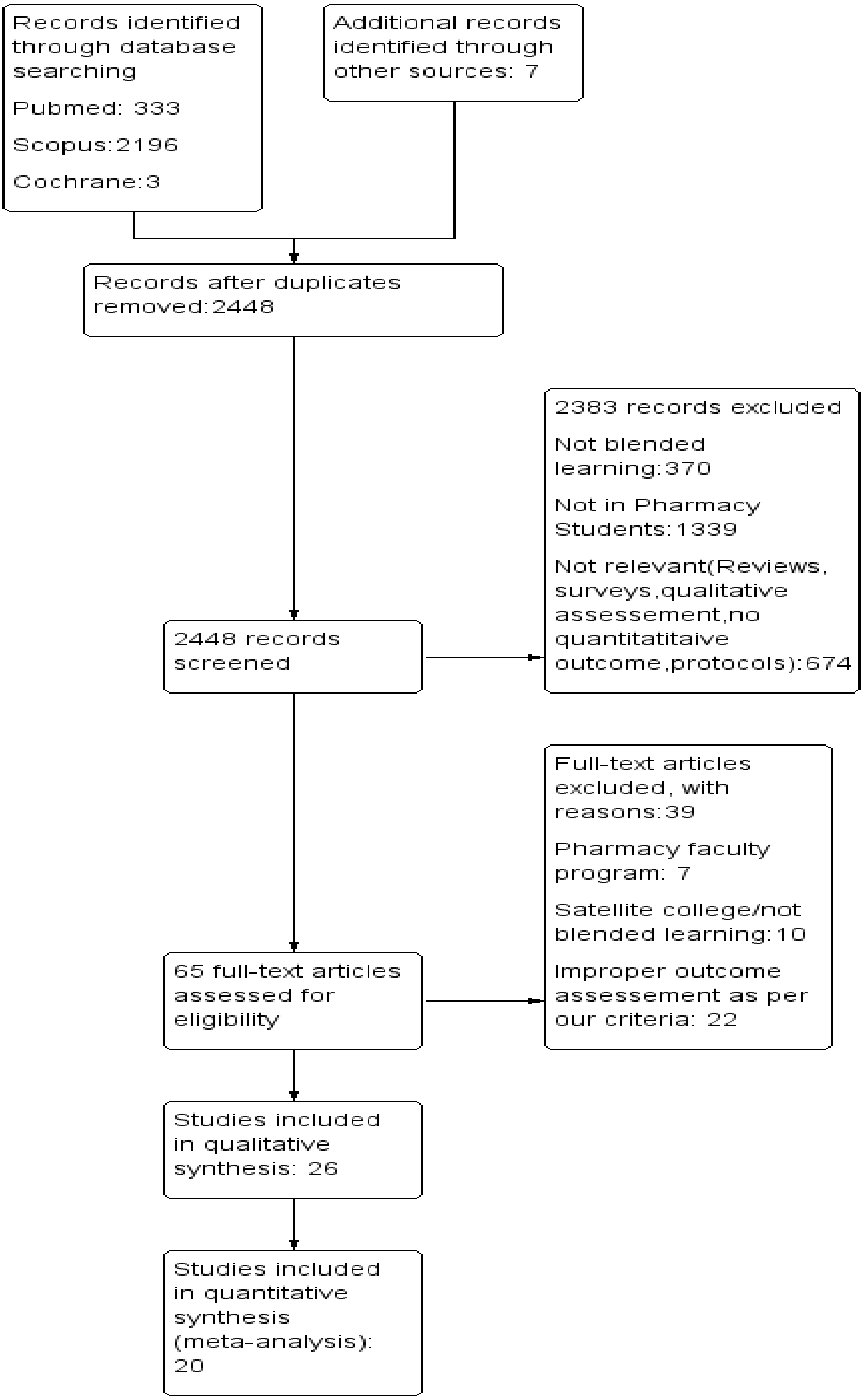

Figure 1: PRISMA FLOW DIAGRAM

Figure2 Click here to access/download;Figure;Figure 2.docx

Study or Subgroup	Experimental Mean	Experimental SD	Experimental Total	Control Mean	Control SD	Control Total	Weight	Std. Mean Difference IV, Random, 95% CI
**1.1.1 cohort studies**								
Cotta, et al., 2016	88.3	9.5	151	84.1	11.3	165	4.9%	0.40 [0.18, 0.62]
David W Stewart et al 2013	72.9	12.63	71	77.15	9.6	65	4.9%	-0.37 [-0.71, -0.04]
Edginton A.N. et al.,2013	78.8	11.7	116	61.8	17.8	109	4.9%	1.13 [0.85, 1.41]
Giuliano C.A. et al.,2016	86.1	21.81	94	75.6	21.81	99	4.9%	0.48 [0.19, 0.77]
Gloudeman et al.,2017	80.5	15.8	102	77.8	16.8	104	4.9%	0.16 [-0.11, 0.44]
Goh C.F. et al.,2019	49.93	25.63	63	41.24	25.63	74	4.9%	0.34 [-0.00, 0.68]
Hughes PJ et al.,2016	88.99	16.46	127	84.87	16.46	121	4.9%	0.25 [-0.00, 0.50]
Kangwantas K. et al.,2017	7.24	1.24	29	6.19	1.76	21	4.6%	0.70 [0.12, 1.28]
Koo C.L. et al.,2016	88.2	7.3	89	83.4	7.9	89	4.9%	0.63 [0.33, 0.93]
Lancaster,2011	84.09	43.87	97	65.15	43.87	97	4.9%	0.43 [0.15, 0.71]
Lockman K. et al.,2017	82.3	10.25	162	77.23	12.43	156	4.9%	0.44 [0.22, 0.67]
McLaughlin JE et al.,2014	82.74	6.67	162	80.03	7.33	153	4.9%	0.39 [0.16, 0.61]
Nazar H et al.,2018	82.2	6.3	69	84.2	6.8	63	4.9%	-0.30 [-0.65, 0.04]
Newsom L et al.,2019	85.8	7.7	153	77.6	13.3	175	4.9%	0.74 [0.52, 0.96]
Prescott WA et al., 2016(2)	80.5	9.6	130	73	12	126	4.9%	0.69 [0.44, 0.94]
Prescott WA et al.,2016(1)	80.6	14.3	131	74.5	12.1	122	4.9%	0.46 [0.21, 0.71]
Wong etal.,2014(1)	88.3	1.9	101	84.1	1.9	103	4.9%	2.20 [1.85, 2.55]
Wong etal.,2014(2)	89.6	2	101	56.8	2.2	103	3.1%	15.54 [13.99, 17.08]
Wong etal.,2014(3)	89.2	1.4	101	73.7	2.1	103	4.1%	8.64 [7.75, 9.53]
**Subtotal (95% CI)**			**2049**			**2048**	**90.3%**	**1.41 [0.94, 1.87]**
Heterogeneity: Tau² = 1.02; Chi² = 857.35, df = 18 (P < 0.00001); I² = 98%								
Test for overall effect: Z = 5.90 (P < 0.00001)								
**1.1.2 RCT**								
He Y.,et al.,2019	88.21	5.95	81	80.05	5.59	56	4.8%	1.40 [1.02, 1.78]
JE McLaughlin et al .,2015	80.12	13.57	57	74.76	15.12	59	4.8%	0.37 [0.00, 0.74]
**Subtotal (95% CI)**			**138**			**115**	**9.7%**	**0.88 [-0.12, 1.89]**
Heterogeneity: Tau² = 0.49; Chi² = 14.53, df = 1 (P = 0.0001); I² = 93%								
Test for overall effect: Z = 1.72 (P = 0.09)								
**Total (95% CI)**			**2187**			**2163**	**100.0%**	**1.35 [0.91, 1.78]**
Heterogeneity: Tau² = 0.98; Chi² = 876.35, df = 20 (P < 0.00001); I² = 98%								
Test for overall effect: Z = 6.08 (P < 0.00001)								
Test for subgroup differences: Chi² = 0.86, df = 1 (P = 0.36), I² = 0%								

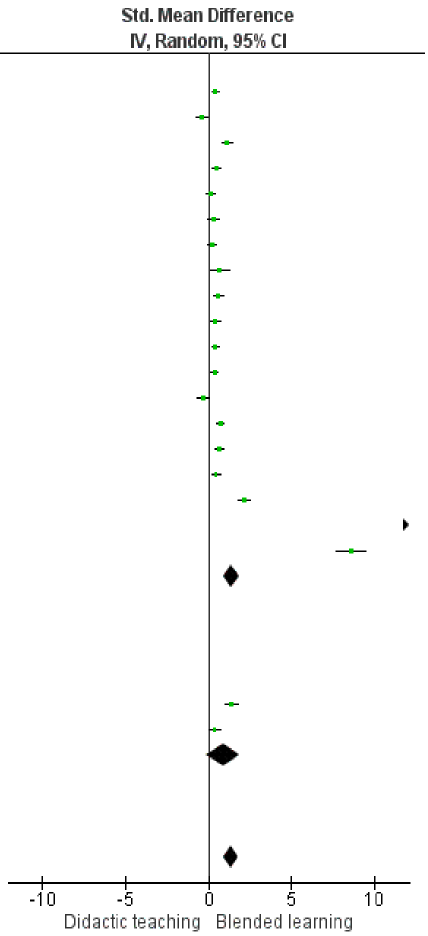

Figure 2: Efficacy of BL vs. traditional teaching in improving knowledge. If more than one topic was delivered by BL in same study (Prescott, Wong) with separate scores for each, we considered them as separate studies (Prescott 1&2, Wong 1, 2&3).

Click here to access/download;Figure;Figure 3.docx

Study or Subgroup	Experimental Mean	Experimental SD	Experimental Total	Control Mean	Control SD	Control Total	Weight	Std. Mean Difference IV, Random, 95% CI
**1.2.1 cohort studies**								
Lockman K. et al.,2017	79.34	9	162	67.01	9.6	156	20.9%	1.32 [1.08, 1.57]
Prescott WA et al., 2016(2)	93.1	7.6	130	89.1	13.8	126	20.9%	0.36 [0.11, 0.61]
Prescott WA et al.,2016(1)	83.5	12.5	131	81.5	12.6	122	20.9%	0.16 [-0.09, 0.41]
Wanat M et al.,2016	87.7	3.7	51	82.6	6.3	54	19.1%	0.97 [0.57, 1.38]
**Subtotal (95% CI)**			**474**			**458**	**81.8%**	**0.70 [0.13, 1.27]**
Heterogeneity: Tau² = 0.32; Chi² = 52.37, df = 3 (P < 0.00001); I² = 94%								
Test for overall effect: Z = 2.39 (P = 0.02)								
**1.2.2 rct**								
Anderson etal.,2017	71.3	14.7	38	61.8	17.7	32	18.2%	0.58 [0.10, 1.06]
**Subtotal (95% CI)**			**38**			**32**	**18.2%**	**0.58 [0.10, 1.06]**
Heterogeneity: Not applicable								
Test for overall effect: Z = 2.37 (P = 0.02)								
**Total (95% CI)**			**512**			**490**	**100.0%**	**0.68 [0.19, 1.16]**
Heterogeneity: Tau² = 0.28; Chi² = 52.46, df = 4 (P < 0.00001); I² = 92%								
Test for overall effect: Z = 2.74 (P = 0.006)								
Test for subgroup differences: Chi² = 0.09, df = 1 (P = 0.76), I² = 0%								

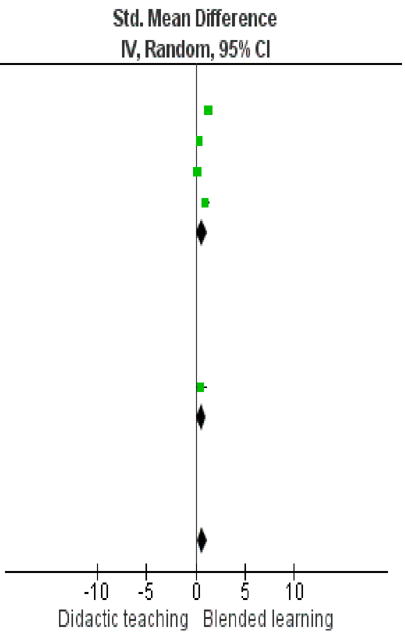

Figure 3: Efficacy of BL Vs. traditional teaching in improving skill. If more than one topic was delivered by BL in same study (Prescott) with separate scores for each, we considered them as separate studies (Prescott 1&2)

Figure4 Click here to access/download;Figure;Figure 4.docx

Study or Subgroup	Experimental Mean	Experimental SD	Experimental Total	Control Mean	Control SD	Control Total	Weight	Std. Mean Difference IV, Random, 95% CI
**1.7.1 cohort studies**								
Cotta, et al., 2016	88.3	9.5	151	84.1	11.3	165	5.5%	0.40 [0.18, 0.62]
David W Stewart et al 2013	72.9	12.63	71	77.15	9.6	65	5.1%	-0.37 [-0.71, -0.04]
Edginton A.N. et al.,2013	78.8	11.7	116	61.8	17.8	109	5.4%	1.13 [0.85, 1.41]
Giuliano C.A. et al.,2016	86.1	21.81	94	75.6	21.81	99	5.3%	0.48 [0.19, 0.77]
Gloudeman et al.,2017	80.5	15.8	102	77.8	16.8	104	5.4%	0.16 [-0.11, 0.44]
Goh C.F. et al.,2019	49.93	25.63	63	41.24	25.63	74	5.2%	0.34 [-0.00, 0.68]
Hughes PJ et al.,2016	88.99	16.46	127	84.87	16.46	121	5.5%	0.25 [-0.00, 0.50]
Kangwantas K. et al.,2017	7.24	1.24	29	6.19	1.76	21	4.1%	0.70 [0.12, 1.28]
Koo C.L. et al.,2016	88.2	7.3	89	83.4	7.9	89	5.3%	0.63 [0.33, 0.93]
Lancaster,2011	84.09	43.87	97	65.15	43.87	97	5.3%	0.43 [0.15, 0.71]
Lockman K. et al.,2017	82.3	10.25	162	77.23	12.43	156	5.5%	0.44 [0.22, 0.67]
McLaughlin JE et al.,2014	82.74	6.67	162	80.03	7.33	153	5.5%	0.39 [0.16, 0.61]
Nazar H et al.,2018	82.2	6.3	69	84.2	6.8	63	5.1%	-0.30 [-0.65, 0.04]
Newsom L et al.,2019	85.8	7.7	153	77.6	13.3	175	5.5%	0.74 [0.52, 0.96]
Prescott WA et al., 2016(2)	80.5	9.6	130	73	12	126	5.5%	0.69 [0.44, 0.94]
Prescott WA et al.,2016(1)	80.6	14.3	131	74.5	12.1	122	5.5%	0.46 [0.21, 0.71]
Wong etal.,2014(1)	88.3	1.9	101	84.1	1.9	103	5.1%	2.20 [1.85, 2.55]
**Subtotal (95% CI)**			**1847**			**1842**	**90.0%**	**0.51 [0.29, 0.74]**
Heterogeneity: Tau² = 0.20; Chi² = 177.76, df = 16 (P < 0.00001); I² = 91%								
Test for overall effect: Z = 4.48 (P < 0.00001)								
**1.7.2 Rct**								
He Y.,et al.,2019	88.21	5.95	81	80.05	5.59	56	5.0%	1.40 [1.02, 1.78]
JE McLaughlin et al .,2015	80.12	13.57	57	74.76	15.12	59	5.0%	0.37 [0.00, 0.74]
**Subtotal (95% CI)**			**138**			**115**	**10.0%**	**0.88 [-0.12, 1.89]**
Heterogeneity: Tau² = 0.49; Chi² = 14.53, df = 1 (P = 0.0001); I² = 93%								
Test for overall effect: Z = 1.72 (P = 0.09)								
**Total (95% CI)**			**1985**			**1957**	**100.0%**	**0.55 [0.33, 0.77]**
Heterogeneity: Tau² = 0.21; Chi² = 199.23, df = 18 (P < 0.00001); I² = 91%								
Test for overall effect: Z = 4.96 (P < 0.00001)								
Test for subgroup differences: Chi² = 0.50, df = 1 (P = 0.48), I² = 0%								

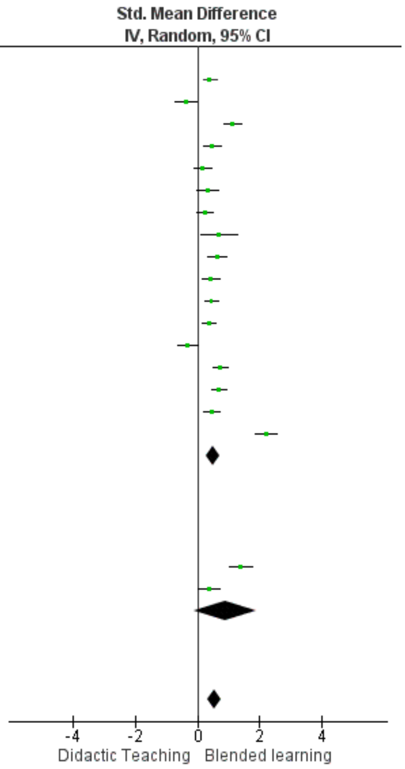

Figure 4. Sensitivity Analysis: If more than one topic was delivered by BL in same study (Prescott, Wong) with separate scores for each, we considered them as separate studies (Prescott 1&2)

 Click here to access/download;Figure;Figure 5.docx

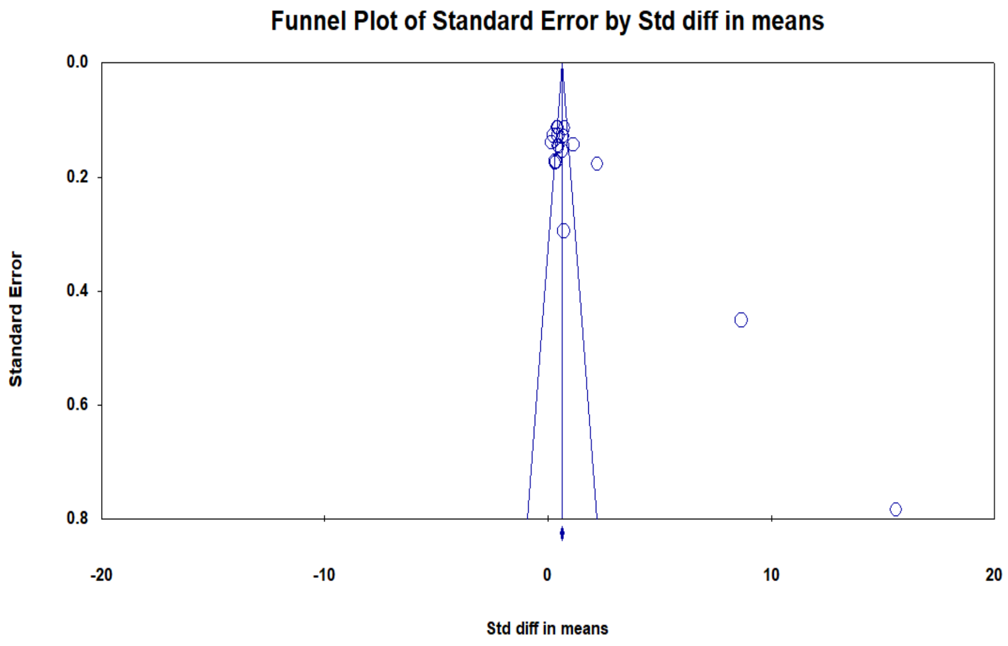

Figure 5: Funnel plot of BL versus traditional teaching in improving knowledge.

Click here to access/download
Supporting Information
Appendices new.docx

TITLE PAGE

Type of paper: Systematic review and meta-analysis

Title of article: Effectiveness of blended learning in pharmacy education: A systematic review and meta-analysis

Running title: Blended learning in Pharmacy education

Authors name & Affiliation:

Athira Balakrishnan[a], Sandra Puthean[b], Gautam Satheesh[b,] M, K Unnikrishnan[b], Muhammed Rashid[a], Sreedharan Nair[a], Girish Thunga[a]

[a]Department of Pharmacy Practice, Manipal College of Pharmaceutical Sciences, Manipal Academy of Higher Education, Manipal, Udupi,Karnataka,India

[b]Department of Pharmacy Practice, National College of Pharmacy, Kozhikode, Kerala, India

Corresponding Author Details:

Dr. Girish Thunga, Assistant Professor, Department of Pharmacy Practice, Manipal College of Pharmaceutical Sciences, Manipal Academy of Higher Education, Manipal, Karnataka-576104, India

Phone number: +91-9880151127; Fax: +91-8202571998

E-mail address: girish.thunga@manipal.edu; ORCID ID: https://orcid.org/0000-0003-1257-969X

EFFECTIVENESS OF BLENDED LEARNING IN PHARMACY EDUCATION: A SYSTEMATIC REVIEW AND META-ANALYSIS

Abstract

Background & Objective: Though blended learning (BL), is widely adopted in higher education, evaluating effectiveness of BL is difficult ~~tough~~ because the components of BL can be extremely heterogeneous. Purpose of this study was to evaluate the effectiveness of BL in improving knowledge and skill in pharmacy education.

Methods: ~~This review follows the PRISMA guideline~~. PubMed/MEDLINE, Scopus and the Cochrane Library were searched to identify ~~the~~ published literature.~~s.~~ The retrieved studies from databases were screened for its title and abstracts followed by the full-text in accordance with the pre-defined inclusion and exclusion criteria. Methodological quality was appraised by modified Ottawa scale. Random effect model used for statistical modelling.

Key findings: A total of 26 studies were included for systematic review. Out of which 20 studies with 4525 participants for meta-analysis which employed traditional teaching in control group. Results showed a statistically significant positive effect size on knowledge (standardized mean difference [SMD]: 1.35, 95% confidence interval [CI]: 0.91 to 1.78, p<0.00001) and skill (SMD: 0.68; 95% CI: 0.19 to 1.16; p=0.006) using a random effect model. Subgroup analysis of cohort studies showed, studies from developed countries had a larger effect size (SMD: 1.54, 95% CI: 1.01 to 2.06), than studies from developing countries(SMD: 0.44, 95% CI: 0.23 to 0.65, studies with MCQ pattern as outcome assessment had larger effect size (SMD: 2.81, 95% CI: 1.76 to 3.85) than non-MCQs (SMD 0.53, 95% CI 0.33 to 0.74), and BL with case studies (SMD 2.72, 95% CI 1.86-3.59) showed better effect size than ~~developing countries (SMD: 0.44, 95% CI: 0.23 to 0.65), non-MCQs (SMD 0.53, 95% CI 0.33 to 0.74) and BL without~~ non-casebased studies (SMD: 0.22, CI: 0.02 to 0.41).

Conclusion: BL is associated with better academic performance and achievement than didactic teaching in pharmacy education.

Keywords: blended learning; flipped learning; education; knowledge; pharmacy; systematic review; teaching

Introduction

Evaluating the effectiveness of ~~B~~blended ~~L~~learning(BL), a thoughtful combination of both online and face-to-face instructions, is tricky because the components of BL can be extremely heterogeneous.[1,2] For instance previous systematic reviews / meta-analyses on BL have included multiple techniques such as virtual face-to-face interaction, simulations, online instruction, e-mails, computer laboratories, mapping and scaffolding tools, computer clusters, interactive presentations, handwriting capture, class room web sites, and virtual apparatuses.[3] Also, there is no standardized proportion in which BL combines online with face-to-face instructions.[4]

Flipped learning 'and 'hybrid learning' are often used interchangeably with BL. In flipped learning, the learner is first exposed to online content, which will be reinforced during face-to-face sessions. [5] Hybrid learning, a combination of face-to-face instruction with computer mediated instruction, is most often used in United States. [6] In all forms of BL, the learner enjoys a certain degree of autonomy in deciding the pace of learning. However, previous reported systematic reviews on BL have not taken the keyword "flipped" in their search strategy [7,8]

Increased research has been published on BL in medical education over last decades. For instance, Quian Liu et al's systematic review and meta-analysis reported that BL has consistent positive effects in comparison with no intervention for knowledge acquisition in health

profession [7]. In another systematic review, McCutcheon et al reported a deficit of evidence on implementation of BL in undergraduate nursing education. [9] Most of the published systematic review and meta-analyse~~i~~s in medical education were focused on medical students or nursing students or other healthcare professionals [8,910] There is only one meta-analysis that evaluates the effectiveness of flipped learning in pharmacy education, with a major limitation namely, lack of prospective randomized control trials (RCT) and restrictions to the domain of flipped contexts. [11] Accordingly, we designed our objective to ~~quantitatively synthesise the studies that~~ assess the effectiveness of BL which employed a combination of online and face-to-face instruction in blended, hybrid and flipped contexts in pharmacy education. We have considered BL as a combination of online and ~~face to face~~face-to-face instruction, excluding other computer mediated forms like virtual labs, gamifications, simulations to limit heterogeneity and included all possible synonyms of blended, hybrid, flipped learning and pharmacy education.

Materials and Methods

This study followed Preferred Reporting Items for Systematic Reviews and Meta-Analyses (PRISMA) Guidelines (PRISMA Checklist attached in data appendix S1)

Eligibility Criteria

We employed PICOS (population, intervention, comparison, outcome, and study design) framework for the inclusion of studies. Studies were considered eligible, if they: (1) were conducted among pharmacy students, (2) used a BL intervention in the experimental group, (3) used traditional lecture based learning as control for two arm studies and pre-test score for single arm studies (4) reported knowledge score/ objective structured clinical examination (OSCE) score as outcome (5) were two groups controlled studies (randomised/non-randomised)/ single group pre-test-post-test studies.~~.~~

We excluded studies which did not explicitly state components of BL i.e. face-to-face learning and computer assisted learning. Computer assisted learning can be any form of technologies like online learning, e-learning, video podcasts, or the application of university learner management system for posting lectures. We excluded studies which employed "virtual face-to-face" interactions (as practiced by universities with satellite campuses). Studies which did not report quantitative outcome of knowledge (comparison of students who completed and did not complete online module, number of correct answers between the groups, comparison of pass percentage), studies which evaluated only online component of BL, and surveys were also excluded. ~~Studies comparing performances of batches~~Multi-year studies ~~–~~without differentiating between study term years~~–~~were excluded. Reviews, short communication, conference proceedings, editorials, meeting abstracts and non-English studies were also excluded.

Data Sources and literature search:

A literature search employing PubMed, Scopus and Cochrane Library, was performed using a comprehensive search strategy since the inception of each database up to mid-December 2020. We employed all the MesH terms and key words for "BL" (Blended learning, blended course, blended program, hybrid learning, hybrid Course, Hybrid Program, Hybrid training, Flipped learning, Flipped Course, Flipped Program, Computer-aided learning, Computer-assisted learning, Integrated learning, Distributed learning, Distributed education Integrated instruction, Computer-aided instruction, Computer-assisted instruction) and "Pharmacy Student" which was obtained from the databases and previous studies. We employed the asterisk (*) as a wildcard character in keyword searches. We also searched for additional reference materials by consulting the cross references listed in the included publications, in addition to Google and Google Scholar. (Details in data appendix S2)

Study selection and Data Extraction

The retrieved studies from databases were screened for its title and abstracts followed by the full-text in accordance with the pre-defined inclusion and exclusion criteria. (List of excluded studies provided in Data appendix S3). We compiled and collated data in a comprehensive data extraction form containing characteristics such as, author and year of publication, population, duration and subject covered, nature of BL, sample size, outcomes. The above data extraction form was perfected by trial and error, by piloting on 3 articles. Three independent reviewers were involved in study selection and data extraction to limit the bias and any disagreements were resolved through consensus or by discussion with another member of research team.

Quality Assessment

Modified Newcastle Ottawa scale (Newcastle Ottawa scale-education) was used to appraise methodological quality of included studies. [12,13,14] This tool assesses the following criteria: 1) representativeness of intervention group (1 point) 2) selection of comparison group (1point) 3) comparability of comparison group (2 point) 4) study retention (1 point) 5) blinding of assessment (1 point), totalling a maximum of 6 points. Two independent reviewers were involved to appraise the methodological quality to limit the bias and any disagreements were resolved through consensus or by discussion with another member of research team.

Data Synthesis

The evidence were synthesised narratively and presented in tabular form. We employed meta-analysis whenever possible. We omitted studies from data pooling whenever data did not meet the requirements of meta-analysis, such as, participant number, mean and standard deviation [SD]. All comparisons were based on scores of consecutive years. If more than one topic was delivered by BL in same study with separate scores for each, we considered them as separate

studies. Review Man 5.3 was used to conduct the meta-analysis.[15] The data were used as mean with SD and outcomes were presented as standardised mean difference (SMD) along with 95% confidence interval (CI). Studies that did not report a SD, the corresponding SD from the p- values and standard errors were generated as per Cochrane guideline [16]. Heterogeneity was assessed by I^2 statistics and random effect model used for statistical modelling. Subgroup analysis were performed to find out potential source of heterogeneity based on factors like studies with case studies and without case studies, studies which reported outcome as a measure of multiple choice questions(MCQs) or non MCQs, and studies from developed countries and developing countries. Sensitivity analysis were performed to ensure the robustness of findings.

Publication bias

We employed a funnel plot for visual inspection of publication bias, which was assessed for statistical significance by Egger's and Begg's test.[16]

Results

A total of 2539 records were retrieved -first, at start, of which 2448 underwent initial screening. Further Next 2383 studies were omitted, finally yielding 65 full-text studies, of from which 26 studies were included for systematic review, wherein and –20 were considered for meta-analysis. (See figure 1 for details of study selection)

Characteristics of studies included for systematic review

Of the 26 studies included, only two employed single arm pre-test-post-test design.[17,18] The remaining 24 studies were controlled studies[19-42] out of which 19 used examination scores of previous year[19-34, 36-41] and one used examination score of subsequent year as control.[35] There were 3 randomised trials[19,14,31] out of which one was cluster randomised.[24] Another study

divided learning materials into didactic and BL in same population.[28] 18 studies originated from USA and 8 studies from other countries.[17,20-21,23-25,26,28,33] (See table 1 for characteristics of included studies)

Table 1: Characteristics of included studies

Author	Country	Population	Topic(duration)	Intrv. details	Study design	Sample size		Post-intrv. academic outcome	Other activities	Mean Result Score		Major outcome
						Intrv.	***Control***			***Intrv.***	***Control***	
Wilson et al [40] (2019)	USA	2nd year pharmaco-therapy students	Selected self-care pharmaco-therapy(NC)	Online (Vimeo) + class activities	Cohort (compared with previous year students' score)	N/A	N/A	Exam performance (65% course grade)-Assessment questions	TBL	83.5%	83.3%	No statistically significant differences in student outcomes
Newsom et al[34] (2019)	USA	1st year students enrolled in spring 2015-17. (Control: spring '14)	Pharmaco kinetics(NC)	Traditional class and video podcast	Cohort Intrv.: 2015-17 Didactic teaching: 2014	2015: 153 2016: 152 2017: 153	2014: 175	Final exam score: questions based on Bloom's taxonomy.	Case based practice problems	2015: 85.8(7.7) 2016: 85.1(9.2) 2017: 78(12)	2014: 77.6 (13.3)	Final exam scores were significantly higher in spring '15 and '16 compared to '14 ($p<0.001$). 2017 scores were similar to that of 2014.
GoH et al[23] (2019)	Malaysia	2nd year Dosage Form II course	3 credit course. Dosage form II(NC).	Pre-recorded video + F2F sessions	Cohort: Two group comparison ('16 & '17 batch)	63	74	Final Exam score. Subjective (5 MCQ + 2 essay)	Online games	49.93	41.24	Final exam performance significantly higher in the flipped classroom group
He et al[24] (2019)	China	Junior year pharmacy under-graduates	Pharma-ceutical marketing (4 months)	Online + class	Cluster randomization	81	56	Final score (subjective – short answer, essays, MCQ)	Case discussion Group activity	88.21 ± 5.95	80.05 ± 5.59	Compared with LBL methods, implementing the FC model improved student performance.
Kouti et al[28] (2018)	Iran	Pharmacy Students (2015-16 batch)	Non-prescription drugs (1 semester)	Electronic based + lecture based	Propective comparative study – 3-group study (f2f, Electronic, BL)	57	-	Final exam score (not clear)	Case studies	E-learning group: 16.17 ± 0.33; Lecture group: 13.75 ±	-	BL method and an e-learning approach can positively influence students' knowledge towards non-prescription drugs

										0.16; BL: 16.39 ± 0.19		
Kangwantas et al[26] (2017)	Thailand	2nd year pharmacy students	Fundamental nutrition (1 year)	Videos (moodle platform) +class activities	Cohort compared with previous year score.	29	21	Pre and post-test within the group Post-test: main exam scores. (subjective-MCQ + short answer)	Case discussion	Pre-test: (7.45±1.89 and Post-test: (8.17±1.44) not statistically different (p =0.08).	Flipped class scored higher (7.24±1.24 vs. 6.19±1.76) (p =0.028)	Student performance as measured by final scores of the module was better than those for the same module taught with a traditional lecture in previous year
Koo et al[27] (2016)	USA	2nd year PharmD students	Pharmaco-therapy (1 year)	Online + F2F discussion	Cohort-comparison (2011 & 2012)	89	89	Exam score: Objective, MCQ	Case study discussion	88.2% (7.3%)	83.4% (7.9%)	The redesigned course improved student test performance and perceptions of learning experience
Giuliano et al[22] (2016)	USA	1st year Pharmacy Students	Drug literature evaluation (1 year)	Youtube lecture + Class session.	Cohort: 2-group study (2013 & 2014)	94	99	Final exam score: subjective-application, analysis & Bloom's taxonomy evaluation	Group activities	86.1%	75.6%	The flipped model is an excellent fit for drug literature content and courses that want to incorporate more active learning
Edginton et al[21] (2013)	Canada	2nd year pharmacy students	Bio-chemistry (1 year)	online + classroom	Cohort: 2011 vs. 2010	116	109	Final grade: subjective-MCQ + calculation + long answer	Group discussion Case studies	78.8 + 11.7	61.8 + 17.8	The student driven BL model correlated positively with increased interest and perceived and actual learning gains. P<0.00001.
Pierce [36](2012)	USA	Pharmacy students	Renal pharmaco-therapy (8 weeks)	Video podcast + classroom	Pre-test and post-test-within the group (2012) Only post-test between	71	N/A	Objective-MCQ	Case discussion	Pre-test (33.5 ± 11.6 and post-test (79.2 ± 10.6): within the group	77.7 ± 4.7	Implementing a flipped classroom to teach renal pharmacotherapy resulted in improved student performance

					groups (2012 & 2011)					Between groups: 81.6 ±4.4		and favourable student perceptions
McLaughlin et al[31] (2015)	USA	PharmD students	Neuro-logic pharmaco-therapy(NC).	e-learning + class	Randomized (same class)	57	59	Final exam score: 9 final exam questions-not clear whether questions are MCQ/subjective.	Case studies	80.12 + 13.57	74.76 + 15.12	Interactive online preparatory tool improves student learning in neurologic pharmacotherapy.
Wong et al [41] (2014)	USA	1st year pharmacy students	Cardiac arrhythmia (3 classes)	Pre-recorded video + class	Cohort: compared with previous year (2012 & 2011)	101	103	Final exam score: 5-6 MCQ on cardiac arrhythmias.	Case based exercises	Basic science: 88.3±1.9 Pharmacology: 89.6±2 Therapeutics: 89.2±1.4	84.1 ± 1.9; 56.8 ± 2.2; 73.7 ± 2.1	Use of the flipped teaching in a 3-class pilot on cardiac arrhythmias improved exam scores for pharmacology and therapeutics classes.
Anderson et al[19] (2017)	USA	1st year pharmacy students	Pharma-ceutical calculations (6 weeks)	recorded lecture + video	Randomized	38	32	Final exam-Skill: OSCE Score at 6 weeks	Case studies	71.3 (14.7)%	61.8 (17.7)%	Average OSCE performance was be higher in flipped model than lecture model
Cotta et al[20] (2016)	Georgia	1st year pharmacy students	Pharma-ceutical calculation (10 weeks)	Pre-recorded video + class	Cohort: 2012 vs. 2011	151	165	Final exam part 2 score-objective graded quizzes	-	88.3 (9.5)	84.1 (11.3)	Flipped classroom can improve student performance and satisfaction in pharmaceutical calculations (P<0.001)
Lancaster[29] (2011)	USA	2nd PharmD	OTC, medicines (15 weeks)	Pre-recorded video + class	Cohort: 2008 vs. 2009	97	97	Final Exam scores-objective-9 quizzes	Clinical based cases with Group discussion Puzzles,	84.09	65.15	Students performed significantly higher on quizzes and examinations when using this hybrid teaching model.

Formatted: Left

									Think pair share activities.			
Stewart[38] (2013)	USA	3rd year pharmaco-therapy	Pharmaco-therapy(NC)	Podcast + active learning	Cohort: 2009 vs. 2010	71	65	Final exam score: 20 MCQ	Group discussion	72.9+1.5 (12.63)	77.15+1.2 (9.6)	The class averages on the final exams were significantly higher for 2009 compared to 2010 batch (P: 0.019).
Lockman et al[30] (2017)	USA	1st year pharmaco-logy & therapeutics course	Pain manag-ement module(NC)	E learning + in class lecture	Cohort: 2015 vs. 2016	162	156	OSCE: skill Knowledge-MCQ	Cases & mini cases, quiz games, Mind-mapping debates	MCQ: 82.30% (SD 10.25) OSCE: 79.34(9)	77.23% (SD 12.43 OSCE: 67.01(9.6)	Student performance improved significantly after flipping the content of pain management module.
Nazar et al[33] (2018)	Qatar	Stage 2 Pharmacy under-graduate students	Pharmacy law(NC)	Online class + In-class activities.	Cohort study: compared with previous year	69 (2016-17)	63 (2015-16) 37 (2014-15)	Final summative examination score	Group discussion	82.2 (6.3)%	84.2 (6.8)% 83.0 (7.6)%	Examination performance appeared to be unaffected by the change in teaching style
Hughes et al[25] (2016)	USA	P1 pharmacy students	Drug information (5 weeks)	Narrated video + F2F lab session	Cohort study: compared with previous years (2012 Vs. 2013)	127	121	Final exam score: Objective-40 MCQ	-	88.99%	84.87%	Mean final exam scores significantly increased ($p < 0.05$)
Gloudeman et al[42] (2017)	USA	1st year pharmacy students	Pharmaceutical calculation (6 week)	Online video + classroom session	Cohort study: compared with previous years (2015 Vs. 2014)	102	104	Final exam score: 13 pharma-ceutical calculation questions	-	80.5 $\pm$ 15.8%	77.8 $\pm$ 16.8%	The mean exam scores of the intrv. were not significantly different than the control (p= 0.253)
Czepula et al[17] (2017)	Brazil	Under-graduate bachelor's	Pharma-ceutical care(NC)	F2F + distance learning	Quasi-experimental, prospective.	Pharma-ceutical care 1: 82		Pre- and post-test: 30 MCQ	-	Module 1: Mean scores increased		Positive results were observed regarding the students'

		degree in pharmacy			Two groups. Both groups received BL of pharma-ceutical care 1 & 2.	Pharmaceutical care 2: 51				from 4.8 to 6.3 (p<0.05) Module 2: Mean scores increased from 4.1 to 5.5 (p<0.05)		performance in the two disciplines
Prescott et al[37] (2016)	USA	1st and 2nd year pharmacy students	Two course: PA1 and PA2(NC)	Online videos + class	Cohort study: Comparison of traditional and BL of PA1 & PA2 (2014-15 vs, 2013-14)	PA1: 130 PA2: 131	PA1: 126 PA2: 122	Final examination score: 20 short answer questions	Group discussion (TBL) Case based learning	Knowledge: PA1: 80.5 (9.6) PA2: 80.6 (14.3) P<0.001 Skill: PA1: 93.1 (7.6) PA2: 83.5 (12.5)	Know-ledge: PA1: 73(12); PA2: 74.5(12.1) P<0.001 Skill: PA1: 89.1 (13.8) PA2: 81.5 (12.6)	BL was associated with improved academic performance and was received by students.
Wanat et al[39] (2016)	USA	3rd year pharmacy students	. Critical care 2hr credit course(4 weeks)	Video recorded lecture + in-class activities	Cohort study: Compared with previous year score. (2013, 2014 Vs. 2012, 2011)	51	54	Overall exam performance: subjective: online quiz + skills: examining patients	Group learning	87.7% (3.7)	82.6% (6.3)	Exam scores of students in BL group is significantly higher than control
Phillips et al[35] (2016)	USA	1st and 2nd year PharmD students	EBM & Therapeutics(6 month)	Online video prior to class room	Cohort study: Knowledge compared with previous year scores - two group comparison.	EBM: 201 Therapeutics: 199	N/A	Final Exam score/-not clear whether questions are MCQ/subjective.		EBM:83% Therapeutics: 97	EBM:85% Therapeutics: 98%	Use of the BLE did not seem to have an impact on long-term knowledge in this study

Hess et al[18] (2016)	USA	2nd year pharmacy students	Patient centred communication skills(One semester,)	Online modules + small group discussion	Single group study	57	-	Pre-test and post-test<br><br>OSCE	Group discussion	Significant Increase in scores from pre-test to post-test.7 domains of pre and posttest scores provided.		Patient-centred interprofessional communication skills improved significantly with BL
McLaughlin et al[32] (2014)	USA	1st year pharmaceutics students	Pharmaceutics course(NC)	Flipped classroom (iLAMSsS + F2F)	Cohort study: Traditional vs. Flipped 2012 vs. 2011	162	153	Final exam grade. Subjective-quizes+examination scores	-	Final score; 165.48 ± 13.34	160.06 ± 14.65	Higher final exam grades in flipped classroom

Intrv.: Intervention; BL: Blended learning, -EBM: Evidence-based medicine; TBL: Team Based Learning; F2F: Face-to-Face, N/A-not Available, NC-not clear MCQ: Multiple choice questions, OSCE: Objective structured clinical examination, PA1: Patient assessment 1 course, PA2: Patient assessment 2 course, iLAMS.: integrated learning accelerator module

Outcome measured

Only 3 studies[18,19,39] reported outcome as skills(patient centred interpersonal communication skills, students' performance on pharmaceutical calculation, and critical care therapeutics) while 21 studies reported only knowledge score. [17, 20-29, 31-36, 38, 40-42] Two reported both knowledge and skills as outcomes.[30, 37] Outcomes were measured variably as mean examination percentage (n=16) or mean examination score (n=6) or objective structured clinical examination (OSCE) (n=2). Two studies reported both examination percentage and OSCE score.

BL approaches

Two studies employed face-to-face session followed by online activities [17, 34] while all other studies employed face--to--face session after watching online content. Only one study reported time spent and workload associated with BL.[37]

Quality assessment of included studies

As per modified Ottawa scale requirements, we ascertained that intervention groups in all the included studies were representatives of target population. Out of 26 studies, 19 used previous year students' score as control, one used subsequent year score as control and 3 studies were randomized. Two studies used analysis of covariance(ANCOVA) for controlling covariates in final analysis [23 ,38] and one used linear regression.[22] In five studies there were no statistically significant differences in students demographics / pre-test (Grade Point Average) between groups by t-test. [27, 30, 32, 34, 41] However, modified Ottawa scale requires controlling for subject characteristics by statistical covariate analysis. Outcome assessment was blinded for 11 studies, as assessor cannot be influenced by group assessment (third party statistician) or assessments did not require human judgments (MCQs/ graded performance). [17,

[19-20, 25, 27, 29-30, 36, 38, 40-41] As all studies were part of curriculum in educational institution, there is no mention about drop outs. All studies obtained a score below 4 except one.[19] (See data appendix S4)

Quantitative analysis

We included 20 studies with 4525 participants for meta-analysis that employed traditional teaching in the control group and had no missing data.

Efficacy of BL versus. Traditional teaching in improving knowledge

Pooled effect of 18 studies showed that knowledge improved significantly in BL, with large effect compared to didactic teaching ((SMD 1.35, 95% CI-0.91 to 1.78, $p<0.00001$). In the knowledge domain, randomised controlled studies had a lower pooled effect (SMD 0.88) than cohort studies (SMD 1.41). There was significant statistical heterogeneity among studies (I^2=98%, $p<0.00001$) with individual effect sizes ranging from −0.37 to 15.54. (See Figure 2)

Efficacy of BL versus traditional teaching in improving skill

Pooled effect size (SMD 0.68, 95% CI: 0.19 to 1.16,Z=2.74,p=0.006) of 4 studies in improving skills, showed statistically significant moderate to large effect, compared with didactic teaching. Significant statistical heterogeneity was observed among studies (I^2=92%, $p<0.00001$). (See figure 3)

Subgroup Analysis

Subgroup analysis of cohort studies, in the knowledge domain, demonstrated advantage for BL over traditional teaching, in developed countries (SMD 1.54, 95% CI 1.01- 2.06) than developing countries (SMD 0.44, 95% CI 0.23-0.65). Studies which employed MCQ scores as outcome showed larger ~~more~~ effect size (SMD 2.81, 95% CI 1.76-3.85) than non MCQs (SMD

0.53, 95% CI 0.33 -0.74). Also, studies which employed case studies/case discussion favoured BL (SMD 2.72, 95% CI 1.86-3.59) than studies without non-case based studies (SMD: 0.22, CI: 0.02 to 0.41). Subgroup analyses of studies improving skill were not performed, as all studies originated from United States of America and all employed case studies/case discussion. (See table 2)

Table 2: : Subgroup Analysis of Cohort studies

Study Characteristics:	Sample size	Test for heterogeneity			Test for effect	
		I^2(%)	Q statistics	P value	Pooled effect size(SMD(C1))	P value
1. Country						
Developed	3731	98	854.67	P<0.00001	1.54(1.01,2.06)	P<0.00001
Developing	366	0	0.89	P=0.35	0.44(0.23,0.65)	P<0.0001
Total	4097	98	857.3	P<0.00001	1.41(0.94,1.87)	P<0.00001
2. Outcome assessment						
MCQ	2002	99	796.46	P<0.00001	2.81(1.76,3.85)	P<0.0001
Non MCQ	1635	76	29.47	P<0.0001	0.53(0.33,0.74	P<0.0001
Not clear	460	96	24.89	P<0.00001	0.23(-0.80,1.25)	0.66
Total	4097	98	857.35	P<0.00001	1.41(0.94,1.87)	P<0.00001
3. Case studies						
Present	2364	99	736.66	P<0.00001	2.72(1.86,3.59)	P<0.00001

Absent	1733	75	31.53	P<0.0001	0.22(0.02,0.41)	0.03
Total	4097	98	857.36	P<0.00001	1.41(0.94,1.87)	P<0.00001

MCQ: Multiple choice questions; SMD: Standardised mean difference; CI: confidence interval

Formatted: Font: 11 pt

Sensitivity Analysis

A sensitivity analysis was performed in studies improving knowledge by removing two studies (Wong et al., [2&3]) which are having lesser weight (3.1% and 4.1%, respectively), and higher outlier (MD: 15.54 and 8.64, respectively) which supported the main results ~~also resulted a similar improved findings in blended learning group, compared to control group, however the score~~ (SMD: 0.55; 95%CI: 0.33 to 0.77) ~~was different from the original findings~~. The result of sensitivity analysis is depicted in Figure 4.

Publication bias

Visual inspection of funnel plot revealed an obvious asymmetry, demonstrating possible publication bias. This was confirmed by Egger's (P=0.00006) and Begg's (P=0.04) test. (See figure 5)

Discussion

This systematic review and meta-analysis primarily attempted to evaluate the impact of BL approach on various outcomes in pharmacy education. We identified 26 studies relevant for systematic review, in which 18 demonstrated significant improvement in learning outcome, against controls. Two of them were single arm studies which also showed improved performance after intervention. 24 of the 26 studies included in this systematic review were

controlled, among which majority (n=19) employed examination scores of previous year(s) as the control. All studies employed first online review of contents followed by face-to-face discussion except two. Studies which employed face-to-face discussion followed by online activities also favoured BL. [17, 34] Face- to- face discussion part of BL in all included studies involved either reinforcing the concepts by tutor or using learning strategies such as case studies, case discussion or group activities.

In addition to the general scarcity of literature comparing BL and traditional methods, a major limitation of the previous meta-analysis by Gillette et al., was the lack of prospective RCTs**.** [11] Our meta- analysis included 20 of the studies included in the systematic review. Our review included 3 RCTs, all of which showed major improvements in either knowledge score or skill. We report a large pooled effect size for knowledge and a medium to large for skills. These findings were statistically significant with high heterogeneity in all analyses and are consistent with those reported by previous meta-analyses in medical education.

Majority of the studies reported knowledge score in terms of either mean examination percentage/score or OSCE, whereas 5 studies reported outcomes based on skill. Many of the studies included in this review also reports that BL has a major effect on improving teaching as well as positive student perceptions about learning. As mentioned earlier, the rich variety of components can attribute to enhanced learning experience as well as increased engagement and learning activities such as group assessment, assessment quizzes and peer discussions. Even the studies that did not report significant difference in acquisition of knowledge– such as those by Phillips et al., and Gloudeman et al. showed that the perceptions of both students and faculty favoured BL. [35, 42]

Another important finding is that BL modules which employed case studies/discussions or case-based scenarios reported better outcomes. A few studies also concluded that positive results obtained may not be attributed entirely to the suggesting that case studies need to be

included in learning strategies. [24, 37] There is evidence to show that case studies simulate real world situations and enhance interactive student-centred learning, particularly in the health professions. Incorporating case studies in a real-world context is extensively useful in pharmacy education, as it enhances students' complex decision-making abilities.

Out of 26 studies, only 4 originated from developing countries, possibly because of poor online connectivity, lack of resources, fear of adopting unfamiliar technology, lack of skill development program to instructors, interruption in power supply and internet connections, affordability, low bandwidth and trust deficit. [20, 17, 26, 28]. A single study that compares time budgets reported that BL techniques were completed ahead of allotted time.[35] BL approach appears to significantly improve the learning outcomes in pharmacy students and reason could be following,

(i) **Relaxed/flexible scheduling**: BL allows students to view electronic materials at their own pace and time

(ii) **Improved interaction**: BL makes classroom discussion more meaningful because of content familiarity.

(iii) **Variety of components**: BL incorporates a rich variety of face-to-face and online components.

This study has a few limitations. Firstly, the search was restricted to the publications in English language, which might have contributed to missing out eligible studies in non-English speaking countries. However, a comprehensive search in various databases would have covered the maximum quality publications. Secondly, our review also excludes conference proceeding and unpublished or grey literatures. However, this may increase the credibility of our findings obtained from full length papers by avoiding the irrelevant or incomplete acquisition of the data. Thirdly, there was high heterogeneity among the outcomes or measures of outcome,

thereby restricting our choice exclusively to studies reporting quantitative outcomes. Fourth, the heterogeneous administration pattern of BL was an another challenge in this review, so we included those studies which used online teaching along with face-to-face approach, this made our result more robust and conclusive. Statistical heterogeneity was high in all analysis. However, this is in accordance with other meta-analysis in medical education [7,8,43] Subgroup analyse~~i~~s did ~~could~~ not find any source of ~~for~~ heterogeneity . Despite the effective search strategy, one major limitation is the ~~at~~ majority i.e. 18 of the 26 studies, were from the US, which could impact the global representativeness of the ~~review~~ findings. Therefore, future research should address the impact of BL in diverse populations from other countries.

Publication bias ~~i.e. the likelihood of positive results being published more frequently than negative results,~~ was addressed by including the three major scientific databases (Pubmed, SCOPUS and Cochrane) during the literature search. This resulted in increased number of papers which may have further increased the likelihood of selecting papers with negative results. In our review, 5 of the 26 studies reported that BL yields either equal or poorer outcomes than didactic teaching.[42, 33, 35, 38, 40]

Conclusion

BL is associated with better academic performance and achievement than didactic teaching in pharmacy education. The COVID-19 pandemic ~~crisis~~ is radically reshaping education sector to transform from conventional teaching to more online learning. In this scenario, it is critical ~~cardinal~~ to conduct more controlled empirical studies to evaluate the effectiveness of BL. Such research can inform education policies and guidelines to standardise blended learning. ~~as online learning could never replace physical university experience. There is an urgent need of uniform strategy of blended learning across the world in its best possible way for the robust and reproducible conclusions.~~

Declaration

Funding: No funding is received from any sources or funding agency for this study.

Conflicts of interest/Competing interests

Authors report no conflicts of interest to declare. The listed authors are solely responsible for the originality and content of the manuscript. The authors have no relevant affiliations or financial involvement with any organization or entity with a financial interest in or financial conflict with the subject matter or materials discussed in the manuscript. This includes employment, consultancies, honoraria, stock ownership or options, expert testimony, grants or patents received or pending, or royalties.

Availability of data and material

The datasets generated during and/or analysed during the current study are not deposited in any repository and it is available from the corresponding author on reasonable request.

Ethics approval

An ethical approval was not required as the study was not included any patients.

References

1. Graham CR. Emerging practice and research in blended learning. In M. G. Moore (Ed.), Handbook of distance education. New York: Routledge.2013:333-350

2. Dziuban C *et al*. Blended learning: the new normal and emerging technologies. ETHE. 2018; 15(1): 3.

3. Means B *et al.* The effectiveness of online and blended learning: A meta-analysis of the empirical literature. *Teach. Coll. Rec.* 2013;115(3): 1-47.

4. Garnham C. Introduction to hybrid courses. Teaching Technology Today. 2002;8.

5. DeLozier SJ *et al.* Flipped classrooms: a review of key ideas and recommendations for practice. *Educ. Psychol. Rev.* 2017; 29(1):141-151.

6. O'Byrne WI *et al.* Hybrid and blended learning: Modifying pedagogy across path, pace, time, and place. *J Adolesc Adult Lit*. 2015;59(2):137-140.

7. Liu Q *et al.* The effectiveness of blended learning in health professions: systematic review and meta-analysis. *J Med Internet Res.* 2016;18(1): e2.

8. Vallée A, Blacher J, Cariou A, Sorbets E. Blended Learning Compared to Traditional Learning in Medical Education: Systematic Review and Meta-Analysis. *J Med Internet Res.* 2020;22(8):e16504

9. McCutcheon K *et al.* A systematic review evaluating the impact of online or blended learning vs. face-to-face learning of clinical skills in undergraduate nurse education. *J Adv Nurs.* 2015;71(2):255-270.

10. Rowe M, Frantz J, Bozalek V. The role of blended learning in the clinical education of healthcare students: a systematic review. *Medical teacher*. 2012;34(4):e216-21

11. Gillette C *et al.* A Meta-Analysis of Outcomes Comparing Flipped Classroom and Lecture. *Am J Pharm Educ*.2018; 82(5):6898.

12. Cook DA *et al.* Method and reporting quality in health professions education research: a systematic review. *Med Educ*. 2011;45(3):227-238.

13. Cook DA *et al.* Internet-based learning in the health professions: a meta-analysis. *JAMA.* 2008; 300(10):1181-1196.

14. Cook DA *et al*. Appraising the quality of medical education research methods: The Medical Education Research Study Quality Instrument and the Newcastle-Ottawa Scale-Education. *Acad Med.* 2015;90(8):1067-1076.

15. Collaboration, T. C. (2014). Review Manager. Copenhagen.

16. Higgins JP *et al.* Cochrane handbook for systematic reviews of interventions: John Wiley & Sons.2019.

17. dos Santos Czepula AI *et al*. Active methodology and blended learning: An experience in pharmaceutical care. *Curr Pharm Teach Learn.* 2018;10(1):106-111.

18. Hess R *et al.* Teaching communication skills to medical and pharmacy students through a blended learning course. *Am J Pharm Educ.* 2016;80(4).

19. Anderson HG *et al.* Comparison of pharmaceutical calculations learning outcomes achieved within a traditional lecture or flipped classroom andragogy. *Am J Pharm Educ*. 2017;81(4).

20. Cotta KI *et al.* Effectiveness of flipped classroom instructional model in teaching pharmaceutical calculations. *Curr Pharm Teach Learn.* 2016;8(5):646-653.

21. Edginton AN *et al.* Using student feedback to design a more effective clinical biochemistry course component. *Curr Pharm Teach Learn.* 2013;5(1):23-32.

22. Giuliano CA *et al.* Evaluation of a flipped drug literature evaluation course. *Am J Pharm Educ*. 2016;80(4).

23. Goh CF *et al.* Flipped classroom as an effective approach in enhancing student learning of a pharmacy course with a historically low student pass rate. *Curr Pharm Teach Learn.* 2019; 11(6):621-629.

24. He Y *et al*. The effects of flipped classrooms on undergraduate pharmaceutical marketing learning: A clustered randomized controlled study. *PLOS One*. 2019;14(4), e0214624.

25. Hughes, P. J., Waldrop, B., Chang, J. J. C. i. P. T., & Learning. (2016). Student perceptions of and performance in a blended foundational drug information course. *Curr Pharm Teach Learn.* 2016; 8(3):359-363.

26. Kangwantas K *et al*. Implementing a flipped classroom approach to a course module in fundamental nutrition for pharmacy students. *Pharm. Educ.* 2017; 17.

27. Koo CL *et al.* Impact of flipped classroom design on student performance and perceptions in a pharmacotherapy course. *Am J Pharm Educ*. 2016;80(2).

28. Kouti L *et al*. Comparison of the effectiveness of three educational methods (e-learning, lectures and blended) on pharmacy students' knowledge of non-prescription drugs. *Pharm. Educ.* 2018; 18:197-201.

29. Lancaster JW *et al.* Online lecture delivery paired with in class problem-based learning… Does it enhance student learning? *Curr Pharm Teach Learn*.2011;3(1):23-29.

30. Lockman K *et al.* Improved learning outcomes after flipping a therapeutics module: results of a controlled trial. *Acad Med.* 2017;92(12):1786-1793.

31. McLaughlin JE *et al*. Comparison of an interactive e-learning preparatory tool and a conventional downloadable handout used within a flipped neurologic pharmacotherapy lecture. *Curr Pharm Teach Learn.* 2015;7(1):12-19.

32. McLaughlin JE *et al.* The flipped classroom: a course redesign to foster learning and engagement in a health professions school. *Acad Med.* 2014;89(2):236-243.

33. Nazar H *et al.* A study to investigate the impact of a blended learning teaching approach to teach pharmacy law. *Int J Pharm Pract.* 2019;27(3):303-310.

34. Newsom L *et al.* Implementation and evaluation of problem-based video podcasts in an introductory pharmacokinetics course. *Curr Pharm Teach Learn.* 2019; 11(12):1213-1220.

35. Phillips JA *et al*. Time spent, workload, and student and faculty perceptions in a blended learning environment. *Am J Pharm Educ.* 2016;80(6).

36. Pierce R *et al*. Vodcasts and active-learning exercises in a "flipped classroom" model of a renal pharmacotherapy module. *Am J Pharm Educ.* 2012;76(10).

37. Prescott WA *et al.* Introduction and assessment of a blended-learning model to teach patient assessment in a doctor of pharmacy program. *Am J Pharm Educ.* 2016;80(10).

38. Stewart DW *et al.* An analysis of student performance with podcasting and active learning in a pharmacotherapy module. *Curr Pharm Teach Learn.* 2013;5(6):574-579.

39. Wanat MA *et al.* A critical care hybrid online elective course for third-year pharmacy students. *Am J Pharm Educ.*2016;80(9).

40. Wilson JA *et al.* Flipped classroom versus a didactic method with active learning in a modified team-based learning self-care pharmacotherapy course. *Curr Pharm Teach Learn.* 2019;11(12):1287-1295.

41. Wong TH et al. Pharmacy students' performance and perceptions in a flipped teaching pilot on cardiac arrhythmias. *Am J Pharm Educ.* 2014;78(10).

42. Gloudeman MW *et al.* Use of condensed videos in a flipped classroom for pharmaceutical calculations: Student perceptions and academic performance. *Curr Pharm Teach Learn*. 2018;10(2):206-210.
43. Fontaine G, Cossette S, Maheu-Cadotte MA, Mailhot T, Deschênes MF, Mathieu-Dupuis G, Côté J, Gagnon MP, Dubé V. Efficacy of adaptive e-learning for health professionals and students: a systematic review and meta-analysis. *BMJ open*. 2019 ;9(8): e025252.

Reviewer 1:

Abstract - You can remove "This review follows the PRISMA guideline." - PRISMA is a reporting standard, not a methodology. In the full text, you can certainly say your review adheres to the reporting standards recommended by the PRISMA statement-

Answer:

Corrected as per reviewer

2. Eligibility (Page 3) - Please clarify. You state in (3) "pre-test score for single arm studies" and (5) "were two groups" - so did you included single group papers or not? Based on results, you included 2 single arm studies, so (5) doesn't appear to be an accurate inclusion criteria.

Answer:

Corrected.

We have included interventional studies like single group pretest and post test studies , two group controlled studies(randomized& non randomized). If it is single grouped, we have taken only those studies with pretest and post test); If it is two group studies, didactic teaching as control. 5 points in eligibility criteria is based on PICOS framework.

3. Acronyms - Spell out all acronyms for first use. For all tables, please include the abbreviations and acronym definitions as a key.

Corrected

4. Page 13 - "face-to-face" and "face to face" (be consistent)

Corrected

5. Quality Assessment - A significant majority of studies used prior year scores as control with few studies attempting to control for confounding. Is it appropriate to pool the results of analyses?

We have separately mentioned pooled effect size of studies with historical group as control and studies with RCT design. Based on previous literature, and Cochrane guideline, we understand nothing wrong to pool this way. We pooled both conditions separately to avoid or adjust this dissimilarity or heterogeneity in the form of a subgroup analysis. Also, we presented the result as an overall to know the effectiveness blended learning as whole. As per Cochrane recommendations, all eligible studies can be included in the meta-analysis, regardless of the risk of bias assessment. Indeed, since almost all studies have low score, Cochrane suggests to present an estimated intervention effect based on all available studies, together with a description of the risk of bias in individual domains.

Reference: (Higgins JPT, Green S. Cochrane Handbook for systematic reviews of interventions version 5.1.0.: the Cochrane collaboration 2011)

As per the Cochrane Guideline of systematic review (section 9.6), we need to perform a subgroup analysis by splitting the data according to some specific characters such as participant, intervention or publication characters etc., to explore the effect of that particular factor in the analysis.

As its educational intervention study in pharmacy education, practically it is difficult to conduct prospective controlled trial in same batch of students in same academic institution. That's why most of the studies adopted historical group as control. Only three studies are RCTs. Out of 26 studies, we included 20 studies with no missing data for quantitative analysis. Among them 8 studies controlled confounding variables (please see quality assessment of studies by modified Ottawa scale). However, modified Ottawa scale requires controlling for subject characteristics by statistical covariate analysis. We have assigned "0" score for studies that tried to compare the baseline characteristics (adjusting confounders) by any method other than statistical covariate analysis. In all those studies, there were no statistically significant differences in students demographics / pre-test (Grade Point Average) between groups by t-test also.

6. Unequal weighted test scores - It appears by focusing on the mean difference, you equally weight 1 point on a 100 point exam vs. a 50 point exam, despite the point value being worth twice as much on the latter.

As per the Cochrane guideline, When studies have used different instruments to measure the same construct, a standardized means difference (SMD) may be used in meta-analysis for combining continuous data, hence we used SMD to combine our results. Reference link is provided.

https://handbook-5-1.cochrane.org/chapter_9/9_2_3_2_the_standardized_mean_difference.htm

Reviewer #2:

This study was a SR and MA that sought to assess the effectiveness of blended learning teaching strategies compared to traditional lectures. The study was well done. Methods were clear. Technical writing and grammar need to be revised. Attached is a file with grammatical revisions

Grammatical errors corrected.
